# Mechanochemical Upcycling of Spent Battery Graphite into a Self-Cleaning Adsorbent for Wastewater Treatments

**DOI:** 10.34133/research.1015

**Published:** 2025-11-26

**Authors:** Zhongyi Liu, Qiangwei Li, Qingming Song, Jia Li, Zhenming Xu

**Affiliations:** ^1^ China-UK Low Carbon College, Shanghai Jiao Tong University, Shanghai 200240, People’s Republic of China.; ^2^ School of Environmental Science and Engineering, Shanghai Jiao Tong University, Shanghai 200240, People’s Republic of China.

## Abstract

The cost-effective upcycling method of waste anode graphite (SG) presents an important challenge within the field of spent lithium-ion battery recycling. In this study, waste graphite was recovered from waste lithium-ion batteries by a one-step mechanochemical method and the high-efficiency graphene-based adsorbent OMG17 was synthesized. Following this, titanium dioxide microspheres (TiO_2_) were integrated through electrostatic self-assembly to create an in situ photo-regeneration adsorbent, referred to as OMG17@TiO_2_. The synthesized OMG17@TiO_2_ exhibits a well-developed pore structure, characterized by regular crack-like pores and an abundance of functional groups. Furthermore, OMG17@TiO_2_ was fabricated into a membrane via the vacuum filtration method, enhancing its practicality for collection in water bodies. The results indicate that the adsorption capacities of OMG17@TiO_2_ for methylene blue and rhodamine B reached 673.67 and 966.58 mg/g, respectively, as determined by the Langmuir isothermal model, significantly exceeding the performance of comparable graphitic adsorbents. Additionally, the regeneration efficiency achieved through ultraviolet (UV) irradiation was found to be as high as 70%. In contrast to traditional desorption methods, the in situ photo-regeneration approach offers distinct advantages in preserving the structural integrity of the material, including the maintenance of pore structure and recovery of specific surface area. Through density functional theory calculations and an examination of the adsorption mechanism, it was established that the pore structure and oxygen-containing functional groups are the primary determinants of the adsorption capacity, while the in situ degradation of pollutants within the pores via UV light serves as the principal mechanism for regeneration.

## Introduction

The recycling of lithium-ion battery (LIB) materials is a crucial step in ensuring the sustainability and environmental protection of LIBs [1]. In current industrial recycling processes, waste graphite is often used as a pyrometallurgical reducing agent for LIB recovery [2], which results in significant carbon dioxide emissions. By 2030, spent lithium batteries may generate 873,900 tons of waste graphite [3] and emit 3.2 million tons of carbon dioxide if recovered through pyrometallurgy. Due to the low economic value of the anode graphite found in spent lithium batteries and the complexity of recovery methods [4,5], there has been limited research on the recovery of waste graphite. At the laboratory scale, most research on waste graphite recovery has been conducted by combining hydrometallurgy with heat treatment to effectively dissolve metal ions in waste graphite and restore its structure, which exhibits volume expansion and surface cracks [6–11]. However, this structure also contains abundant oxygen-containing functional groups and a porous architecture, making it advantageous for the upcycling of spent materials. Waste graphite is often upcycled into graphene via the Hummers method [12,13]. Just to recover 1 kg of waste graphite, over 30 kg of H_2_SO_4_, 3 kg of KMnO_4_, and 2 kg of H_2_O_2_ are consumed [14]. This method has significant potential for improvement regarding both environmental and economic benefits. It is crucial to find a cost-effective method to upgrade and recycle waste graphite for greater value.

Adsorbent based on waste graphite is a kind of high-value upcycling [15]. The graphene-based high-performance adsorbent typically interacts through electrostatic forces [16], hydrophobic effects [17], and π–π stacking [18]. However, desorption, which is a critical step in adsorption separation technology [19], poses significant challenges in practice due to the aforementioned strong interactions. Conventional desorption methods typically necessitate the use of substantial quantities of acids [20], bases [21], or organic solvents [22], such as hydrochloric acid (HCl), sodium hydroxide (NaOH), and ethanol. While these reagents facilitate desorption, their application may compromise the structural integrity of the original material [23]. This inherent difficulty in desorption may hinder the broader application of graphene-based adsorbents [24]. Consequently, the development of an environment-friendly, convenient, rapid, and reagent-efficient desorption method remains challenging, particularly for adsorbents exhibiting high adsorption capacities [25,26].

In this study, we present a cheap method for upcycling spent graphite (SG) from waste lithium batteries through a straightforward self-assembly technique combined with physical ball milling. This approach facilitates the development of a high-efficiency adsorbent, which is called OMG17@TiO_2_, enables nonsolvent regeneration, and supports various collectible applications. The adsorption capacities of OMG17@TiO_2_ for methylene blue (MB) and rhodamine B were found to be 673.67 and 966.58 mg/g, respectively, surpassing the values reported in the majority of existing literature. Furthermore, the desorption efficiency of OMG17@TiO_2_ reached 70%, 51% higher than a simple mixture of OMG17 and TiO_2_, with a retention of 60% efficiency after 5 desorption cycles. Notably, a comparison of pore volume and specific surface area using the Brunauer–Emmett–Teller (BET) method indicated that the photocatalytic in situ desorption technique effectively restores the original pore volume when contrasted with traditional methods. Density functional theory (DFT) calculations elucidated the influence of oxygen-containing functional groups on OMG17 in the adsorption of MB, revealing that the hydroxyl and carboxyl groups generated through ball milling significantly enhance the adsorption of pollutants.

## Results

### Synthesis and characterizations

**Fig. 1.**
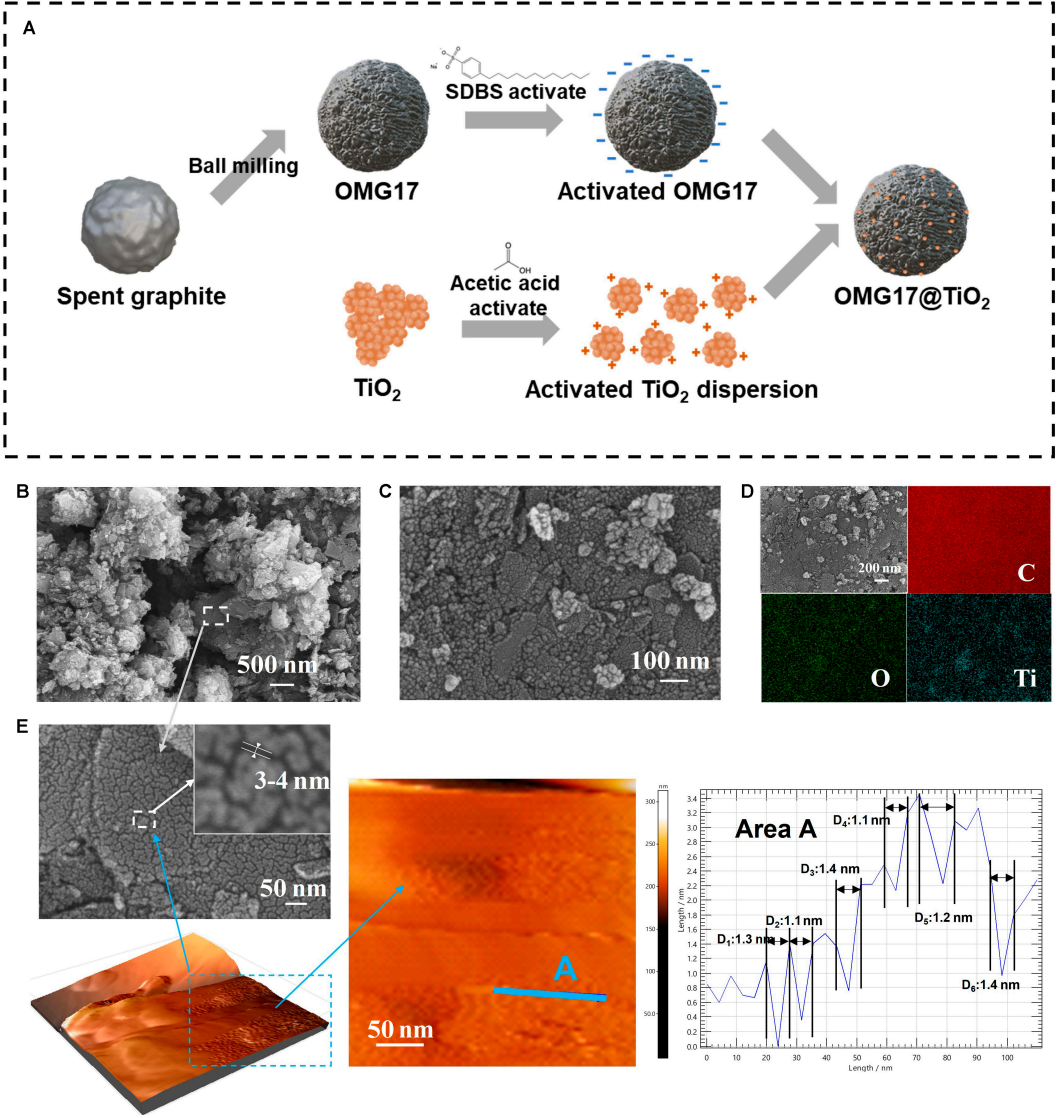
Surface morphology and pore characteristics of materials. (A) Synthetic route to OMG17 and OMG17@TiO_2_. (B) SEM image of OMG17. (C) SEM image of OMG17@TiO_2_. (D) Energy-dispersive x-ray spectroscopy of OMG17@TiO_2_. (E) Atomic force microscope image of OMG17 and SEM image of pore structure of OMG17.

OMG17 was prepared through high-energy ball milling of purified SG (Table S1 and Fig. S1) under the optimized condition (Tables S2 and S3 and Fig. S2). OMG17@TiO_2_ was synthesized through electrostatic self-assembly of TiO_2_ and OMG17 as base materials (Fig. S3). The detailed synthetic route is shown in Fig. 1A. The morphology of OMG17@TiO_2_ and OMG17 is observed in scanning electron microscopy (SEM) images as shown in Fig. 1. OMG17 shows a porous structure formed by the interconnection of nanospheres and particle size reduction in contrast with SG. Notably, Fig. 1E shows that the surface of OMG17 has a uniform crack-like structure, and the crack width is maintained at 3 to 4 nm. Atomic force microscopy images show that the crack depth remains at 1 to 2 nm, which may be caused by the internal stress generated by ball milling. For OMG17@TiO_2_, TiO_2_ nanoparticles were uniformly distributed in OMG17 with an average particle size of 20 nm. OMG17@TiO_2_ retains the morphological characteristics of TiO_2_ and OMG17. Mapping shows the overlap of C element and Ti element, which is consistent with the SEM results. The complete and continuous graphite diffraction fringes can be observed in transmission electron microscopy images, which proves the existence of multilayer graphite, and the degree of graphitization is high (Fig. S4).

**Fig. 2.**
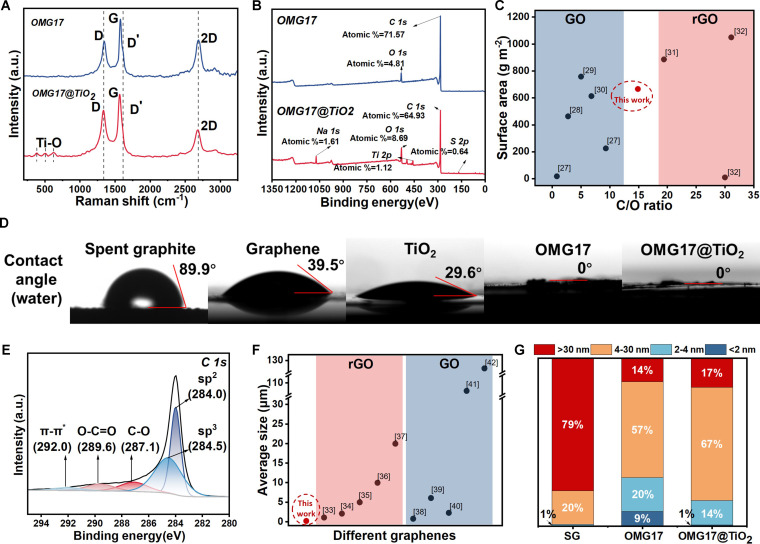
Characteristics of OMG17 and OMG17@TiO_2_. (A) Raman spectrum of OMG17 and OMG17@TiO_2_. (B) XPS survey of OMG17 and OMG17@TiO_2_. (C) Comparison of the carbon to oxygen ratio (C/O) of different graphene [27–32]. (D) Static water contact angle of different materials. (E) XPS C 1s spectra of OMG17 and OMG17@TiO_2_. (F) Comparison of the size of different graphene [33–42]. (G) Pore volume distribution with pore size of SG, OMG17, and OMG17@TiO_2_.

OMG17 showed specific graphenization characteristics through high-energy ball milling. As exhibited in Fig. 2A, a symmetrical Gaussian peak appears at 1,610 cm^−1^, while the 2-dimensional peaks are gradually blue-shifted and symmetrical, indicating the production of thin-layer graphene. X-ray diffraction (XRD) (Fig. S5) shows that the diffraction peak appears at the 2-theta angles of 23°, which also indicates the appearance of graphene. Notably, as shown in Fig. 2B and C, we compared the carbon to oxygen ratio (C/O) of different graphene species by the 6-area integration of the x-ray photoelectron spectroscopy (XPS) peaks of C and O, showing that OMG17 exhibits a high specific surface area compared to similar materials, as well as a notable difference in oxygen content between graphene oxide (GO) and reduced GO (rGO). The oxygen-containing functional groups of OMG17 and OMG17@TiO_2_ (Fig. S6) are formed when the edge carbon defects generated during high-energy ball milling are exposed to oxygen, mainly carboxyl and hydroxyl groups, as shown in Fig. 2E. Compared with the Hummers method, Fig. 2F shows that the graphene produced by high-energy ball-milling method is smaller in size. At this C/O ratio and particle size, OMG17 exhibits superhydrophilicity and excellent dispersion in water compared to other carbon-based materials shown in Fig. 2D. The surface oxygen-containing functional groups increase the hydrophilicity of OMG17, making it easier to disperse in aqueous solutions, increasing the contact probability with pollutants and increasing its adsorption rate. N_2_ adsorption–desorption isotherms are employed to investigate the specific surface area and the pore structures of OMG17@TiO_2_ and OMG17(Fig. S7). The specific surface area of OMG17 calculated by BET surface area analysis is 665.96 m^2^/g, which is much higher than 8.664 m^2^/g of SG. As shown in Fig. 2G, the enormous micropores and large specific surface area in OMG17 can not only supply anchor sites for pollutants but also provide storage spaces.

### Adsorption performance

**Fig. 3.**
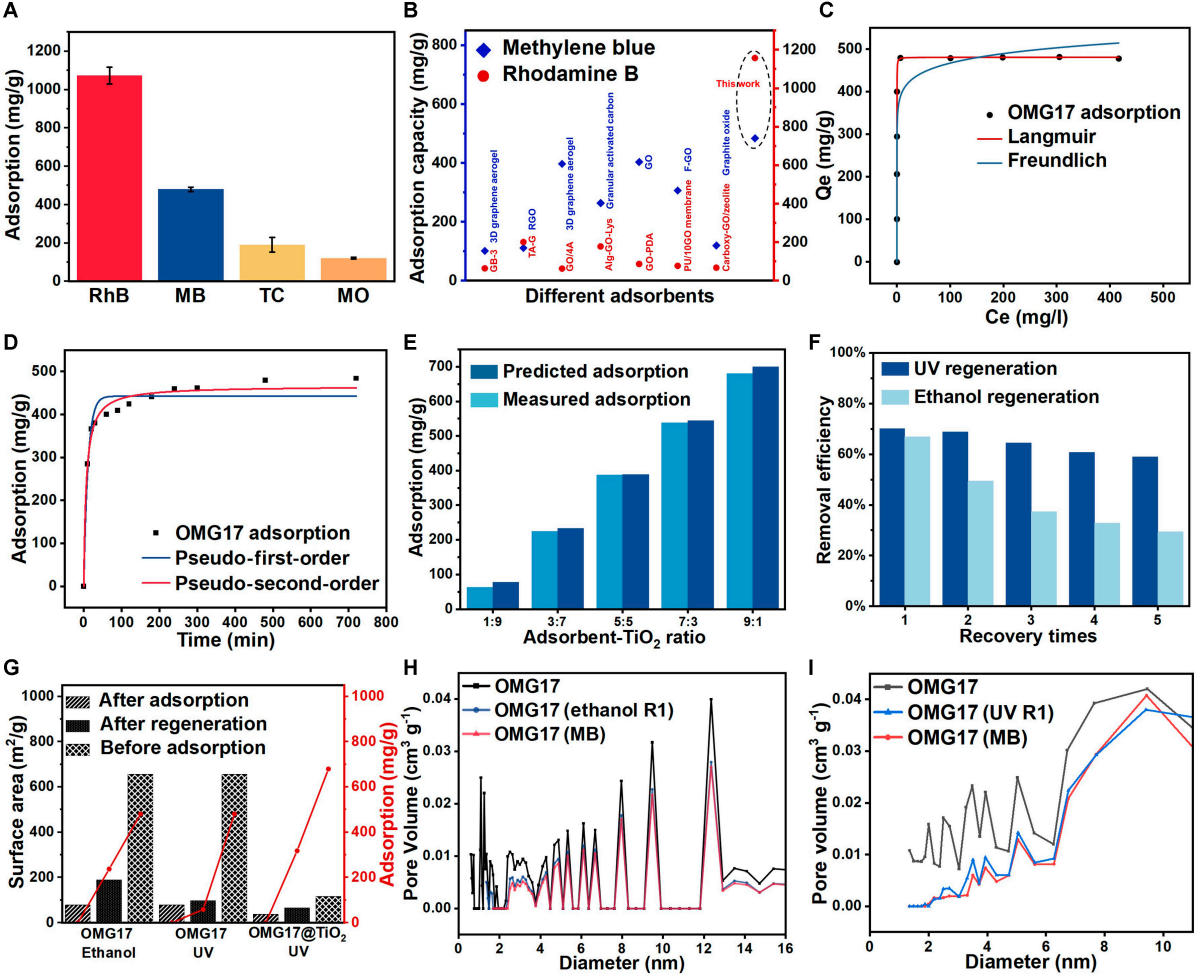
Adsorption and regeneration properties of OMG17 and OMG17@TiO_2_. (A) Adsorption capacity of OMG17 for different pollutants. (B) Comparison of adsorption capacity of OMG17 and different materials [43–57]. (C) Adsorption isotherm models of OMG17. (D) Adsorption kinetic models of OMG17. (E) Comparison of predicted adsorption capacity and measured adsorption capacity after adding different content of TiO_2_. (F) Comparison of UV light and ethanol recycling efficiency. (G) Specific surface area changes with adsorption capacity before and after regeneration. (H) Pore diameter changes after ethanol regeneration of OMG17. (I) Pore diameter changes after UV regeneration of OMG17@TiO_2_.

To test the adsorption performance, different pollutants were used to test the adsorption properties of OMG17 as shown in Fig. 3A, and the adsorption capacity of rhodamine B and MB reached 1,015.9 and 480.9 mg/g, respectively, which far exceeded the reported graphene-based materials (Table S4) shown in Fig. 3B. The adsorption isotherm and kinetic models of OMG17 under aqueous condition were investigated to explain between adsorbents and MB. As presented in Fig. 3C and D, the Langmuir model describes the monolayer adsorption nature fitted best with *R*^2^ value of 0.98 (Table S5), which implies that OMG17 had a monolayer adsorption mechanism onto homogeneous sorbent surface. The quasi-second-order kinetic process (Table S6) shows that the adsorption amount increases rapidly to 380.27 mg/g in the first 30 min after the beginning of adsorption, and then the rate slows down. The rate constant of the quasi-second-order kinetic equation is 3.23 × 10^−4^ g mg^−1^ min^−1^, which is lower than the values reported in the literature, showing a fast adsorption rate (Table S7). Meanwhile, through the adsorption kinetics and thermodynamics data of rhodamine B, it was found that the adsorption characteristics of rhodamine B are similar to those of MB (Tables S5 and S6 and Fig. S8).

Furthermore, to confirm that the adsorption performance of OMG17 is not affected by electrostatic self-assembly process, the adsorption performance of OMG17@TiO_2_ is explored. The adsorption capacities of OMG17@TiO_2_ for MB and rhodamine B reached 673.67 and 966.58 mg/g, respectively, as determined by the Langmuir isothermal model. Due to the activation of sodium dodecyl benzene sulfonate (SDBS; 95%), OMG17 brings much sulfonyl group (Fig. S9), enhancing the adsorption performance to 777.59 mg/g. At the same time, the adsorption capacity of OMG17@TiO_2_ for MB reached 673.67 mg/g. The kinetic and thermodynamic test results of OMG17@TiO_2_ are similar to those of OMG17, which are both consistent with the Langmuir model and the quasi-second-order kinetic process. According to the adsorption thermodynamics and kinetics, the adsorption process of OMG17 for rhodamine B also follows this rule (Tables S8 and S9 and Figs. S10 and S11). As shown in Fig. 3E, predicted adsorption refers to the sum of the adsorption capacity of OMG17 and TiO_2_ after activation, and measured adsorption refers to the adsorption capacity of OMG17@TiO_2_ after synthesis. The predicted adsorption capacity is slightly lower than the measured adsorption capacity, which may be due to the attachment of TiO_2_ to the pore surface of OMG17, reducing the specific surface area and pore volume (Fig. S12). The above experiments indicate that the electrostatic self-assembly process has little effect on the adsorption properties of OMG17.

### Regeneration performance

In general, graphene-based adsorbents are often regenerated using reagents such as HCl, NaOH, and ethanol. Ethanol was used to perform a cyclic regeneration experiment on OMG17. Figure 3F shows that after the first regeneration, the adsorption capacity of OMG17 was 66.95%, and there was a relatively obvious decrease in the adsorption capacity of OMG17 with each cycle of the experiment. After 5 cycles, the adsorption capacity was only 29.34% of that of the original material. In order to explore the reasons for the degradation of recycling performance, XPS spectra (Fig. S13) and BET were conducted to explain the characteristics changes of OMG17. The functional groups on the surface of OMG17 have almost no change after the generation. The semiquantitative analysis of its constituent elements by XPS shows that the oxygen content on the surface of OMG17 increases with the number of regenerations. One possible explanation is that when elution with ethanol is employed, some hydroxyl or ethanol molecules may adhere to the OMG17 surface or become embedded in its pores, resulting in an increase in its oxygen content. This accumulation can obstruct the pore structure of the adsorbent and alter the proportion of oxygen-containing functional groups. It is well known that physical adsorption plays an important role in the adsorption of pollutants from porous carbon materials. Figure 3G demonstrates that the decrease of specific surface area leads to the decrease of adsorption capacity, and the physical adsorption plays the main role. The pore volume distribution (Fig. 3H) showed that the internal pore size was partially occupied after OMG17 adsorbed MB. In the process of ethanol regeneration of OMG17, the original pore structure of MB was occupied by ethanol after elution by ethanol, resulting in the decrease of specific surface area and the inability to recover pore volume. This may be the mechanism that explains the decline in cycle performance.

To avoid the structural destruction of adsorbent materials in the cycle, we prepared OMG17@TiO_2_ with self-cleaning function by in situ photodegradation. OMG17@TiO_2_ is regenerated in an aqueous solution using ultraviolet (UV) light. After the OMG17@TiO_2_ is adsorbed and saturated, the internal adsorbed pollutants are degraded in situ by UV light for 24 h. Figure 3F shows that after the first in situ photo-regeneration, the adsorption capacity of OMG17@TiO_2_ was 70%. At the same time, by comparing its re-adsorption capacity with the materials reported in other studies, it is proven to be superior (Table S10). To verify the sustainability of this method, we uninterruptedly saturated OMG17@TiO_2_ adsorption and performed in situ photolysis. After 5 trials, the adsorption capacity of OMG17@TiO_2_ remained at about 60% of the original capacity. This is a better cycling performance of our prepared method using in situ photolysis compared to the traditional method. In order to investigate the mechanism, we performed a BET test to probe the changes in the physical adsorption of the material. Figure 3I shows that in the 2- to 13-nm pore size range, the pore volume of OMG17@TiO_2_ was first shrunk due to the adsorption of MB and then partially restored due to in situ photolysis. Unlike Fig. 3H, where conventional methods may change the material structure due to the involvement of other solvents, in situ photolysis has been in an aqueous environment where the intensity of UV light is not strong enough to change the porous features and surface functional groups of the OMG17 substrate material (Fig. S14), resulting in a much smaller impact on its surface structure.

### Investigation of adsorption and regeneration mechanism

**Fig. 4.**
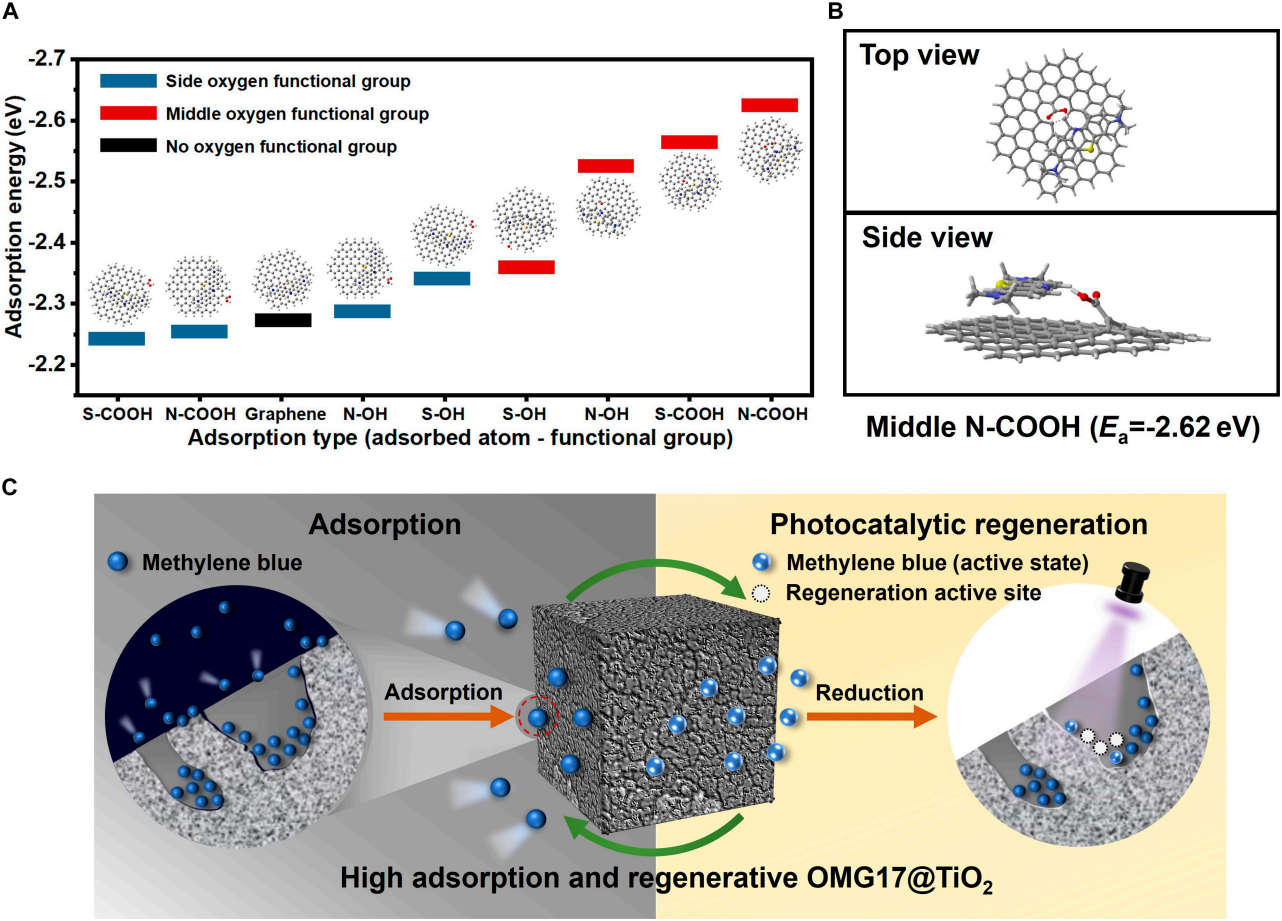
(A) Adsorption energy for the adsorption of methylene blue (MB) by a monolayer of graphene influenced by the functional groups and their positions. (B) Lowest adsorption energy (*E*_a_) for MB on graphene with a single oxygen-containing functional group and corresponding adsorption model. (C) Mechanism of OMG17@TiO_2_ in situ photocatalytic regeneration process.

To elucidate the action site and adsorption mechanism of OMG17 in the removal of MB, DFT calculations were conducted. Based on the spatial arrangement of oxygen-containing functional groups in OMG17 and the adsorption positioning of MB relative to OMG17, several potential adsorption models can be proposed. Specifically, the adsorption of sulfur or nitrogen atoms from MB occurs when graphene is functionalized with hydroxyl or carboxyl groups. Furthermore, the positioning of the oxygen-containing functional groups—whether at the center or edge of the graphene structure—must also be considered. The primary mechanisms facilitating the chemisorption of MB onto graphene include electrostatic interactions, π–π stacking, hydrogen bonding, and covalent bonding. In constructing the adsorption model, we selected a parallel orientation between graphene and MB, as this configuration enhances the π–π stacking interactions. Figure 4A illustrates the adsorption energies associated with various adsorption models. The adsorption energy of intact graphene bound to MB was −2.27 eV, while the adsorption energies of other graphene variants containing functional groups were lower than this baseline value, except for graphene with edge carboxyl groups. This reduction in energy can be attributed to the formation of electrostatic interactions or hydrogen bonds between the hydroxyl and carboxyl groups and MB. However, the presence of edge carboxyl groups causes the graphene to become more curled, thereby reducing the π–π bonding interactions. From the perspective of functional group location, the lowest adsorption energies for the central hydroxyl and carboxyl groups were 0.32 and 0.37 eV lower than those of the peripheral hydroxyl and carboxyl groups, respectively. In terms of adsorbed atoms, when considering the adsorption of MB to sulfur and nitrogen atoms on graphene, graphene generally exhibits a stronger affinity for nitrogen atoms. Additionally, from the standpoint of functional groups, the carboxyl group has a greater influence on adsorption energy than the hydroxyl group. In addition to chemisorption, physical adsorption emerges as a significant mechanism due to the high specific surface area and abundant pore structure of OMG17. SEM analysis (Fig. S15) reveals that the particle size of MB adsorbed on the surface of OMG17 predominantly ranges from 3.06 to 5.1 nm, which coincides with the substantial pore volume of OMG17 within the 2- to 14-nm range. This characteristic contributes to the material’s robust adsorption capacity. Based on the aforementioned DFT calculations and experimental findings, the superior adsorption performance of OMG17 can be attributed to several factors: (a) The high specific surface area and abundant micropore volume provide ample space for MB and other pollutants, resulting in numerous physical adsorption sites; (b) carbon defects and functional groups generated through high-energy ball milling are predominantly located at the edges of the graphene structure, which are more conducive to pollutant adsorption than the central functional groups; (c) the presence of diverse functional groups, such as carboxyl, hydroxyl, and sulfonate, facilitates the electrostatic adsorption of MB and promotes the formation of hydrogen bonds.

The principle of photocatalytic degradation of MB using TiO_2_ has been extensively studied. This degradation process can be categorized into 3 distinct stages: adsorption, photolysis, and degradation. Initially, MB molecules are adsorbed onto the surface of the semiconductor material through either physical or chemical adsorption. Subsequently, upon exposure to UV light, electron-hole pairs are generated within the semiconductor. In the final stage, these electrons and holes participate in redox reactions at the interface, leading to the degradation of MB molecules and the formation of innocuous oxidation products. In our study, experimental evidence demonstrates that titanium dioxide (TiO_2_) effectively catalyzes the degradation of pollutants within the adsorbent. As illustrated in Fig. S16, both OMG17 and the mixture of OMG17 with TiO_2_ exhibit limited catalytic regeneration effects in solution. The combination of TiO_2_ and OMG17 through this method achieves a degradation efficiency of 70%. This phenomenon can be attributed to 2 primary factors: First, the challenge of TiO_2_ catalysis arises from its limited contact with pollutants, as OMG17 tends to adsorb in proximity to TiO_2_. Second, the high conductivity of graphene enhances the separation of electron-hole pairs during the photocatalytic process, thereby improving the efficiency of photogenerated electron transfer. Figure 4C depicts the mechanism underlying the regeneration process of OMG17@TiO_2_. Initially, MB is adsorbed within the material due to the presence of various functional groups and a porous structure on the surface of OMG17. Given that TiO_2_ is uniformly distributed on the internal surface of OMG17@TiO_2_, irradiation with UV light generates electron-hole pairs. Concurrently, MB molecules located near TiO_2_ undergo decomposition via free radical oxidation. Ultimately, the MB adsorbed within the material is decomposed, facilitating the regeneration of the material. In situ photocatalytic regeneration eliminates the need to alter the solvent environment of the adsorbent, in contrast to regeneration using ethanol or HCl. On one hand, the active sites on the adsorbent remain intact, unaffected by changes in pH or the adsorption of other solvents. On the other hand, photocatalytic regeneration is more convenient and rapid, thereby avoiding the costs and environmental pollution associated with solvent use.

### Application

**Fig. 5.**
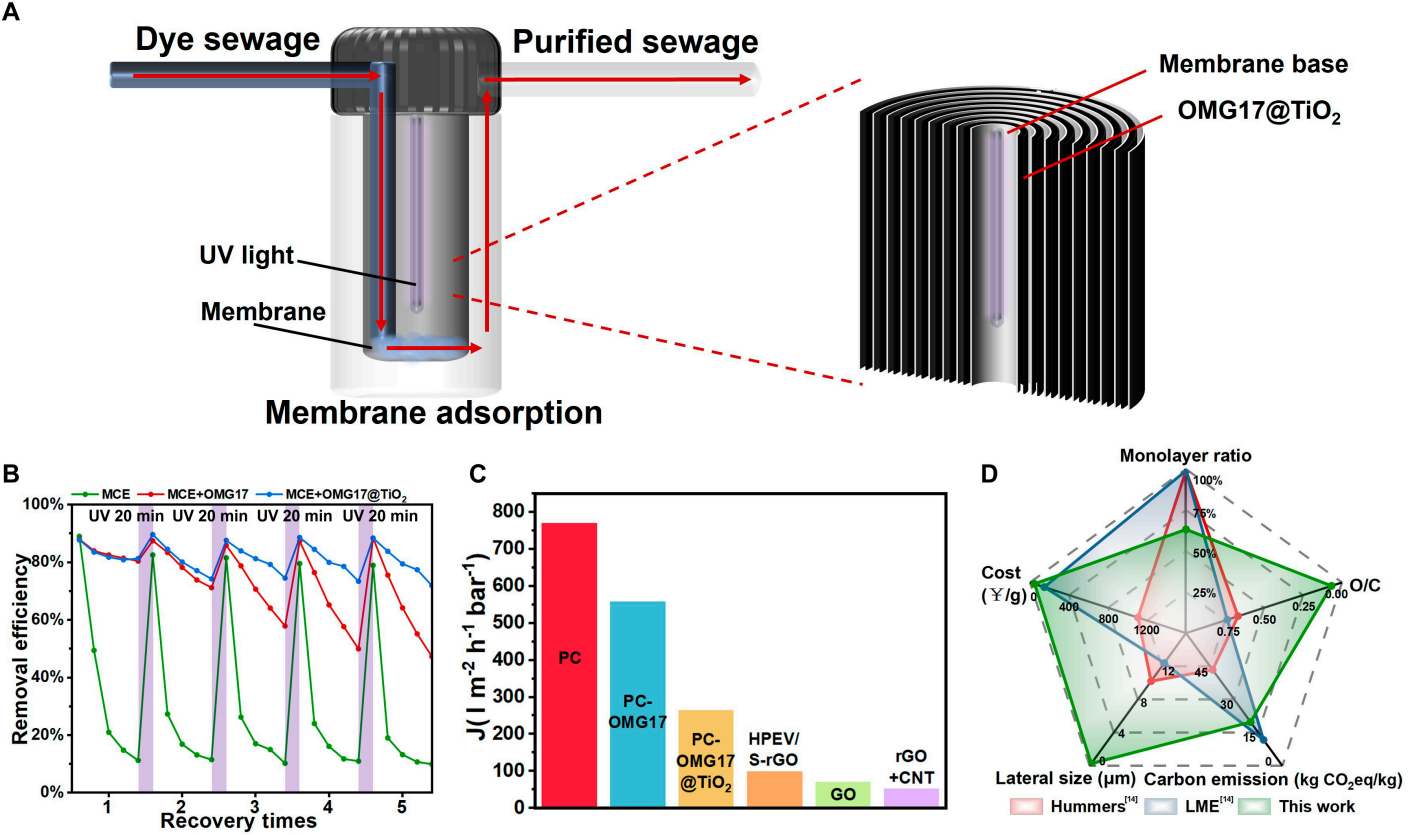
Application of OMG17@TiO_2_ adsorption membrane. (A) Illustration and design of a nonpressurized membrane filter. (B) Water fluxes of membranes [58–60]. (C) Removal efficiency of different membranes. (D) Comparison of Hummers method, liquid membrane electrolysis (LME) method, and ball-milling method in this work [14].

Based on the excellent dye adsorption and cyclic adsorption properties of OMG17@TiO_2_, a kind of recycled dye wastewater membrane was designed for the treatment of pipeline wastewater in a factory of weaving industry. The experimental setup for dye filtration is shown in Fig. 5A and B. The filter consists of 2 sections of pipes, UV lamps, membrane loaded with OMG17@TiO_2_, and a compression device between the pipes (Fig. S17). The membrane was used to adsorb the dye, and the transparent part at the connection of the 2 sections of pipes had a UV lamp irradiating on the surface of the film for membrane regeneration.

Since water flux, inlet and outlet water concentration and serviceable duration together determine the ability of adsorbent materials to treat dye wastewater. As shown in Fig. 5B, we simulated that the feed water containing a mixed solution of 20 mg/l MB flowed through the membrane containing 5 mg of OMG17@TiO_2_ under the action of a peristaltic pump. As a comparison, we also prepared the membrane containing 5 mg of OMG17 and without adsorbents. The efficiency of pollutant removal using the OMG17@TiO_2_ membrane can reach 81.3%, and it remains above 71.8% after 5 cycles. In contrast, the removal rates for the OMG17 membrane and the basement membrane were only 80.4% and 11.1%, respectively, under the same conditions. The OMG17 membrane lacks TiO_2_ material, which is essential for photo-regeneration. After 5 cycles of UV regeneration for 20 min each time, the removal rate of OMG17 film decreased significantly, and the final removal rate was only 47.2%. Additionally, as illustrated in Fig. 5C and Fig. S18, the OMG17@TiO_2_ membrane exhibits an exceptionally high water flux compared to commercially available membranes for mature applications, and it can operate effectively with minimal water pressure.

In order to evaluate the advantages of the industrial process for producing graphene from waste graphite using high-energy ball milling. We compared the industrial production indicators of various graphene preparation methods (Fig. S19 and Tables S11 and S12). The industrial-scale high-energy ball-milling method offers several benefits, including a streamlined process, reduced pollution, minimal reagent use, and the utilization of waste as raw materials. It demonstrates significant advantages over the Hummers method in terms of cost, carbon emissions, oxygen content, and particle size. Additionally, the recently developed liquid membrane electrolysis (LME) methods also present benefits regarding particle size, cost, and oxygen content. Consequently, the high-energy ball-milling method discussed in this paper is both cost-effective and environmentally sustainable for the large-scale recovery of waste lithium battery negative graphite materials.

## Conclusion

In this study, we employ a straightforward and cost-effective high-energy ball-milling technique to recover waste graphite for the preparation of graphene adsorbents. Additionally, we utilize a mild regeneration method to facilitate the efficient recovery of the graphene-based adsorbent. The waste graphite from LIB anodes was processed into the graphene-based OMG17 sample using a one-step ball-milling method, thereby facilitating the value-added utilization of waste graphite. The OMG17@TiO_2_ samples were synthesized through electrostatic self-assembly. OMG17@TiO_2_ exhibits a stable structure, a uniform composite of TiO_2_ microspheres with super-hydrophilicity, abundant oxygen-containing functional groups on the surface, and a consistent crack-like pore structure. The adsorption capacity of OMG17@TiO_2_ for dyes reached 673.67 mg/g for MB and 966.58 mg/g for rhodamine B, significantly surpassing that of similar graphene-based adsorbents. DFT calculations indicated that the adsorption capacity for MB was enhanced by the oxygen-containing functional groups generated during ball milling. After the adsorption of MB, the in situ regeneration efficiency using UV light can reach 70%, demonstrating good cycling performance and superior pore recovery ability compared to traditional regeneration methods. This regeneration process is primarily attributed to the enhanced photocatalytic in situ adsorption of MB within the graphene pores facilitated by the TiO_2_ composite, which effectively releases the adsorption activation sites. The OMG17@TiO_2_ membrane was prepared using a vacuum filtration method, allowing for water flux under normal pressure, exhibiting stability in aqueous environments, and facilitating convenient material collection and application.

## Materials and Methods

### Materials

The spent LIBs used in this research were mainly from waste notebook computers and retired mobile phones. The reagents involved include the following: NaCl (Guaranteed Reagent, 99.8%) was obtained from Aladdin Chemical Reagent Co. Ltd.; MB (Analytical Reagent) was obtained from Shanghai Macklin Biochemical Co. Ltd.; ethanol (Analytical Reagent, 99.7%) was obtained from Aladdin Chemical Reagent Co. Ltd.; rhodamine B (99%) was obtained from Glatt Co. Ltd.; methyl orange (MO, 98%) was obtained from Meryer (Shanghai) Biochemical Technology Co. Ltd.; tetracycline (TC, Chemical Pure) was obtained from Shanghai Macklin Biochemical Co. Ltd.; sodium dodecyl benzene sulfonate (SDBS; 95%) was obtained from Glatt Co. Ltd.; TiO_2_ (P25, 20nm) was obtained from Shanghai Macklin Biochemical Co. Ltd.; l-theanine (98%) was obtained from Shanghai Macklin Biochemical Co. Ltd. Deionized water was used throughout the experiments; acetic acid (99%) was obtained from Sinopharm Chemical Reagent Co. Ltd.

### Pretreatment of spent LIBs

The spent LIBs were dismantled manually (Fig. S1): The batteries were entirely discharged for 24 h within 12 wt % NaCl solution for the safety of the experiment. Afterward, the battery case was removed, the cathode and anode electrodes were separated, and SG was peeled down from the anode current collector. To remove residual lithium and electrolyte, the graphite was washed with deionized water for 3 times and then screened with 120-mesh sieve. Finally, the graphite completely removed the residual electrolyte in the sample for 2 h in N_2_ atmosphere at 500 °C in a tubular furnace.

### Preparation of OMG17

We directly convert SG into OMG17 in one step through high-energy ball milling (Retsch, MM 500 nano). The ball-milling parameters are shown in Table S1. Five grams of the above SG was weighed and put into the stainless steel milling tank (capacity: 50 ml), and 60 g of stainless steel balls (diameter: 5 mm) was added. The milling frequency was set as 28 Hz, and the milling time was set as 48 h. After continuous dry milling, the obtained powders were named OMG17 and then vacuum dried for subsequent use.

### Preparation of OMG17@TiO_2_

OMG17@TiO_2_ is synthesized by electrostatic self-assembly method. Fully dried OMG17 powder (150 mg) was completely dispersed by ultrasound in 100 ml of deionized water. Then, 2.509 g of sodium dodecyl benzene sulfonate was added to the solution. Ultrasonic treatment and stirring were performed to ensure it reacts fully. The activated OMG17 powder was obtained by centrifugation and dried under vacuum. TiO_2_ (2 g) was added to 50 ml of deionized water, and 0.5 ml of acetic acid was added after full mixing. After ultrasonication and stirring, it is dried under vacuum. Finally, the dried TiO_2_ powder and the activated OMG17 powder were placed in 100 ml of pure water at a mass ratio of 1:9, stirred at 70 °C for 4 h, and dried in a vacuum oven at 80 °C to obtain OMG17@TiO_2_.

### Preparation of adsorption membrane

The adsorption membrane was fabricated using a vacuum filtration method. A total of 5 mg of the prepared adsorption material was thoroughly dispersed in 100 ml of water, followed by the addition of 5 mg of l-theanine. The mixture was stirred at room temperature for 6 h. Subsequently, the mixture was ultrasonicated for 1 h. After that, it was vacuum-filtered through either a mixed cellulose ester (MCE) membrane (pore size: 0.45 μm; diameter: 50 mm) or a polycarbonate (PC) membrane (pore size: 50 nm; diameter: 47 mm). Finally, the membrane was dried in an oven at 30 °C to yield the adsorption membrane.

### Characterization

The hydrophobicity of materials is reflected by the contact angle measuring instrument (Drop Shape Analyzer-DSA25, KRÜSS Scientific). XRD (Mini flex 600, Japan) was conducted in the 2θ range of 5° to 90° with a scan rate of 5°/min^−1^. Raman spectra (Renishaw inVia Qontor, UK) were tested in the range of 500 to 3,500 cm^−1^; infrared (IR) spectra were recorded on Nicolet IS5 in the wave range of 400 to 4,000 cm^−1^ through the KBr pellet method; x-ray photoelectron spectra (AXIS UltraDLD) were used for qualitative and semiquantitative detection of chemical components of samples. The surface areas of the sample were obtained by nitrogen adsorption–desorption isotherms using the BET method on BELSORP-MAX, Japan. Material morphology was investigated using a field emission scanning electron microscope (Gemini300, ZEISS, Germany), an energy-dispersive spectrometer (INCA X-Act, Oxford), an atomic force microscope (Gemini 360/Semilab), and a transmission electron microscope (JEOL 2100F). The particle size distribution was measured using a nanoparticle size potentiometer (Zetasizer Nano ZS 90, Malvern Instruments Ltd., UK).

### Pollutant adsorption application experiment

The initial concentration of the pollutant solution was 400 mg/l. First, 15 ml of the pollutant solution and 5 mg of adsorbents were added to a 40-ml capped glass tube. The mixture was then shaken at 250 rpm for 12 h at room temperature (25 °C) using a shaker, followed by filtration through a 0.22-μm filter membrane. The filtrate was collected for subsequent quantitative analysis. Pollutant solution is determined by UV visible spectrophotometry. A UV visible spectrophotometer (Thermo Fisher Scientific Evolution 300 UV-vis Spectrometer) is used to test at the wavelength of 663 nm. The MB adsorption capacity was calculated according to Eq. (1):

$$q_{t}=\frac{\left(c_{0}-c_{t}\right)V}{m}$$ (1)

where *C*_0_ and *C_t_* represent the initial concentration of pollutants (mg/l) and the concentration of pollutants at time *t*, respectively; *V* represents the volume of solution (l); and *m* represents the mass of adsorbent (g).

Herein, we optimized the milling time, milling frequency, and ball to powder ratio in terms of adsorption capacity. The response surface methodology (RSM) is used to complete the optimization design.

### Regeneration performance test

This study investigates 2 types of regeneration experiments: traditional ethanol regeneration and photocatalytic regeneration. Traditional ethanol regeneration involves adding the adsorbent to the pollutant solution until it reaches full saturation, followed by the separation of the adsorbent from the excess pollutant using vacuum-assisted equipment. The filtered adsorbent is then continuously rinsed with ethanol until the rinsed ethanol is colorless. Subsequently, the washed adsorbent is dried in preparation for the adsorption test. In contrast, photocatalytic regeneration entails saturating the adsorbent, followed by pumping and separation. The adsorbent is then mixed with deionized water and subjected to UV light irradiation for a duration of 24 h. After this process, the adsorbent is washed and dried for the adsorption test. The regeneration rate is calculated according to Eq. (2):

$$R_{m}=\frac{q_{r}}{q_{0}}$$ (2)

where $q_{r}$ and $q_{0}$ represent the amount of adsorption after regeneration and the amount of adsorption that has never been regenerated, respectively.

### Evaluation of membrane performance

The performance tests of membrane filtration processes were conducted using stirred cells (Amicon UFSC40001) (Fig. S2). The effective filtration area was 13.4 cm^2^. The deionized water as the feed was pumped into the membrane module, and a transmembrane pressure of 2 bar was controlled by a regulating valve. The performance tests were performed at room temperature. The water permeance ($P_{w}$, l m^−2^ h^−1^ bar^−1^) was calculated using Eq. (3):

$$P_{w}=\frac{∆V}{A\times ∆t\times ∆P}$$ (3)

where ∆V is the volume of penetrant solution, A is the effective membrane area, ∆t is the filtration time, and ∆P is the transmembrane pressure difference.

The osmosis-driven membrane performance tests were carried out using a self-established apparatus. The effective filtration area was 13.4 cm^2^. A 1 mM MB solution is pumped continuously across the adsorbent membrane surface. The liquid was collected every 30 min, and the concentration was tested. The performance tests were performed at room temperature. The pollutant rejection rate (R,%) was calculated using Eq. (4):

$$R=\left(1-\frac{C_{p}}{C_{f}}\right)\times 100\%$$ (4)

where $C_{p}$, and $C_{f}$ are the pollutant concentrations in the permeate and feed, respectively, which were determined by a UV visible spectrophotometer (Thermo Fisher Scientific Evolution 300 UV-vis Spectrometer).

### DFT calculation computational methods

All calculations were carried out with the Gaussian 16 software. The PBE0 functional was adopted for all calculations in combination with the D3BJ dispersion correction. For geometry optimization, the def2svp basis set was used. Visualization is completed using VESTA.

## Data Availability

All data are available in the manuscript or the Supplementary Materials.
