# Supplementary material for: Mechanochemical Upcycling of Spent Battery Graphite into a Self-Cleaning Adsorbent for Wastewater Treatments
Source: Research (Wash D C). 2025 Nov 26;8:1015. doi: 10.34133/research.1015 (PMC12646489; doi:10.34133/research.1015)
Supplement: Supplementary 1 — Supplementary Text Figs. S1 to S19 Tables S1 to S12 [file research.1015.f1.docx]

Supplementary Materials for

**Mechanochemical Upcycling of Spent Battery Graphite into a Self-Cleaning Adsorbent for Wastewater Treatments**

Zhongyi Liu ^1^, Qiangwei Li ^1^, Qingming Song ^2^, Jia Li^1^*, Zhenming Xu^2^

^1^ China-UK Low Carbon College, Shanghai Jiao Tong University; Shanghai, 200240, People’s Republic of China.

^2^ School of Environmental Science and Engineering, Shanghai Jiao Tong University; Shanghai, 200240, People’s Republic of China.

Corresponding author: weee@sjtu.edu.cn

**The file includes:**

Supplementary Text

Figs. S1 to S19

Tables S1 to S12

References

**The influence of impurities in waste graphite.** The waste graphite used in this experiment has undergone multiple processes of acid washing, pyrolysis, and screening, and has fully removed the possible residual metal elements such as cobalt, nickel, iron, etc., as well as organic binders and other factors that may affect the subsequent experiments.

**Table S1.** Element content of waste graphite.

| Elements | C | H | N | S | Co | Cu | Li | Ni |
| --- | --- | --- | --- | --- | --- | --- | --- | --- |
| Content (% in mass) | 96.96 | 0.75 | <0.10 | 0.10 | 0.0163 | 0.0177 | 0.0542 | 0.0005 |


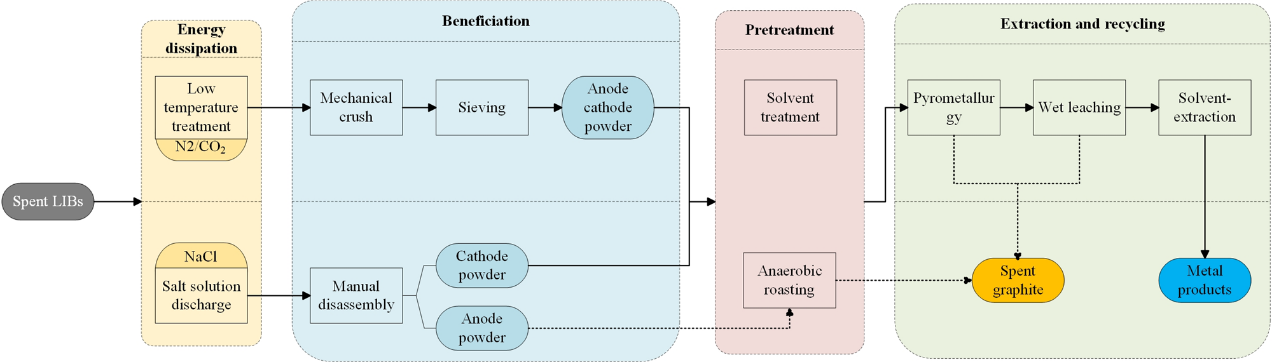


**Figure S1.** Process of recycling spent LIBs by traditional methods (up) and this paper (down).

**Selection of the optimal adsorption material.** According to Table S1 and Figure S2, we select the default quadratic fitting method in the Analysis software to select the optimal adsorption material. Using the Analysis software, conduct a regression analysis on the adsorption capacity data of methylene blue by the OMG series materials. Obtain the 3D corresponding surface graph under the combined effect of ball milling frequency, ball milling time, and ball-to-material ratio, and obtain the optimal solution for the systematic analysis. After data analysis, we defined the optimal ball milling parameters as OMG17.

**Table S2.** Experimental Condition of the Ball Milling Parameters Experiments.

|  | **Milling frequency (Hz)** | **Milling time (h)** | **Ball to powder ratio** | **Adsorption capacity (mg/g)** |
| --- | --- | --- | --- | --- |
| **OMG1** | 20 | 60 | 12 | 445.12 |
| **OMG2** | 20 | 40 | 14 | 427.41 |
| **OMG3** | 20 | 40 | 10 | 337.86 |
| **OMG4** | 20 | 20 | 12 | 261.64 |
| **OMG5** | 24 | 60 | 14 | 472.57 |
| **OMG6** | 24 | 60 | 10 | 406.34 |
| **OMG7** | 24 | 40 | 12 | 427.49 |
| **OMG8** | 24 | 40 | 12 | 413.18 |
| **OMG9** | 24 | 40 | 12 | 420.88 |
| **OMG10** | 24 | 40 | 12 | 418.34 |
| **OMG11** | 24 | 20 | 14 | 356.40 |
| **OMG12** | 24 | 20 | 10 | 216.59 |
| **OMG13** | 28 | 60 | 12 | 464.91 |
| **OMG14** | 28 | 40 | 14 | 473.29 |
| **OMG15** | 28 | 40 | 10 | 417.46 |
| **OMG16** | 28 | 20 | 12 | 368.38 |

**Table S3** Analysis of variance for the fitted models of adsorption capacity**.**

| **Number** | **Time** | **Ratio** | **Frequency** | **Q** | **Desirability** |
| --- | --- | --- | --- | --- | --- |
| **1** | **47.711** | **14.000** | **28.000** | **487.378** | **0.886** |
| 2 | 47.675 | 13.999 | 28.000 | 487.377 | 0.886 |
| 3 | 47.705 | 13.999 | 28.000 | 487.377 | 0.886 |
| 4 | 47.863 | 13.997 | 28.000 | 487.374 | 0.886 |
| 5 | 47.742 | 13.975 | 28.000 | 487.371 | 0.886 |


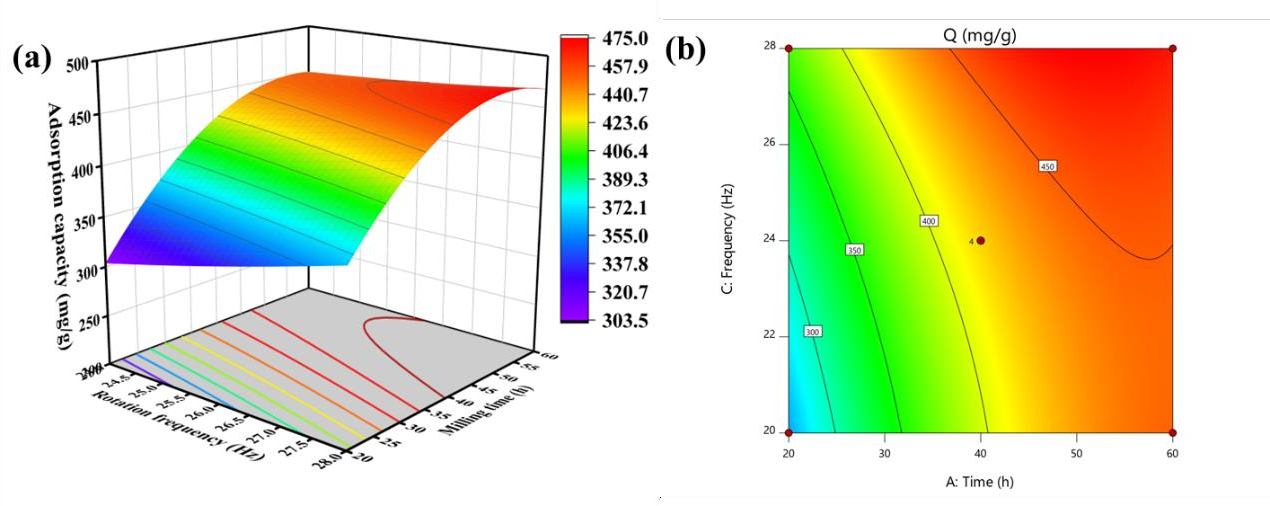


**Figure S2.** (a) Response 3D surface; (b) contour of adsorption capacity: milling frequency and milling time.


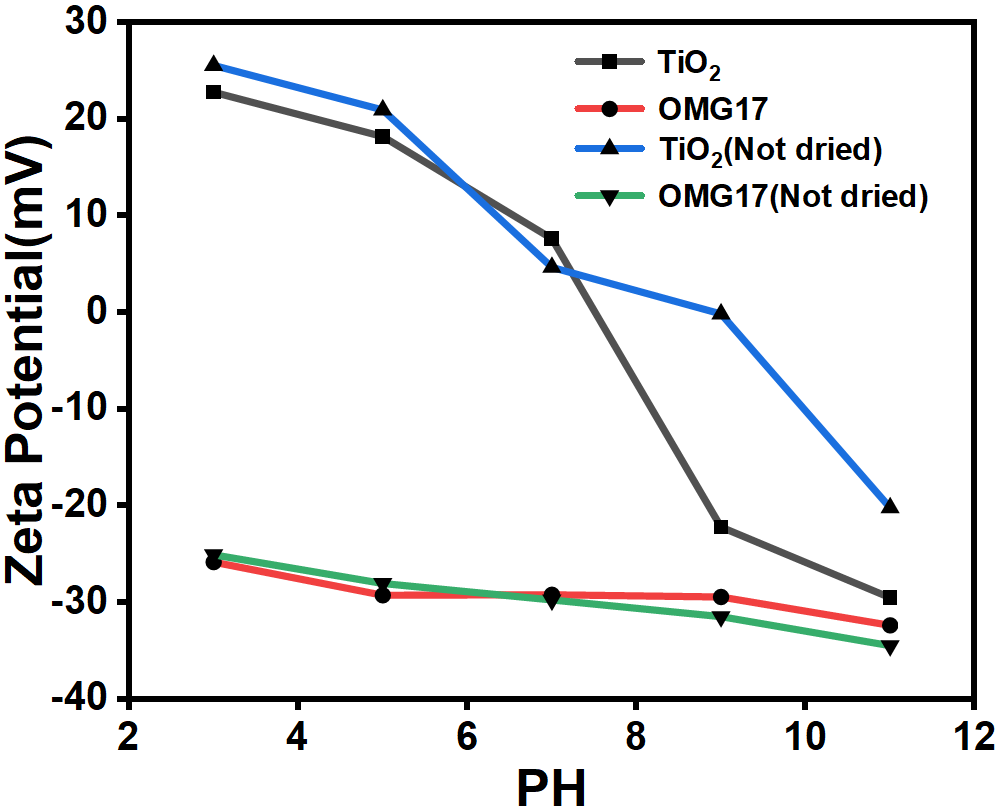


**Figure S3.** The zeta potential of the activated raw materials used in electrostatic self-assembly.


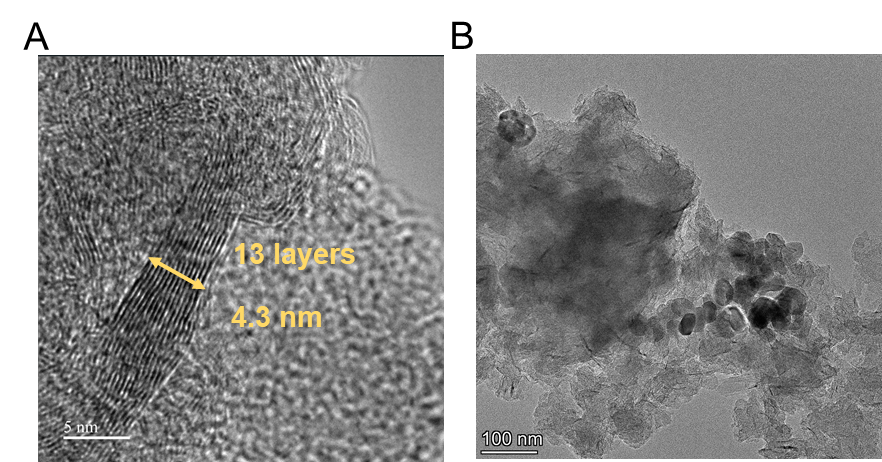


**Figure S4.** TEM of (a) OMG17, (b) OMG17@TiO_2_.


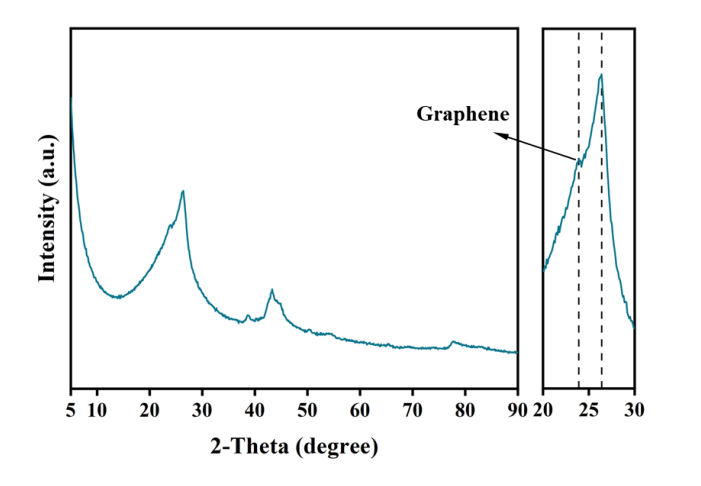


**Figure S5.** XRD pattern of OMG17.


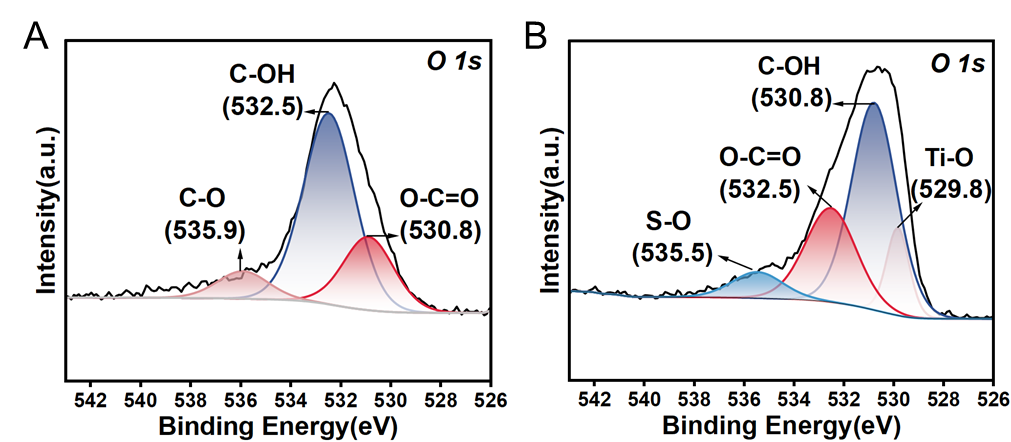


**Figure S6.** (a)XPS O 1s of OMG17. (b)XPS O 1s of OMG17@TiO_2_.


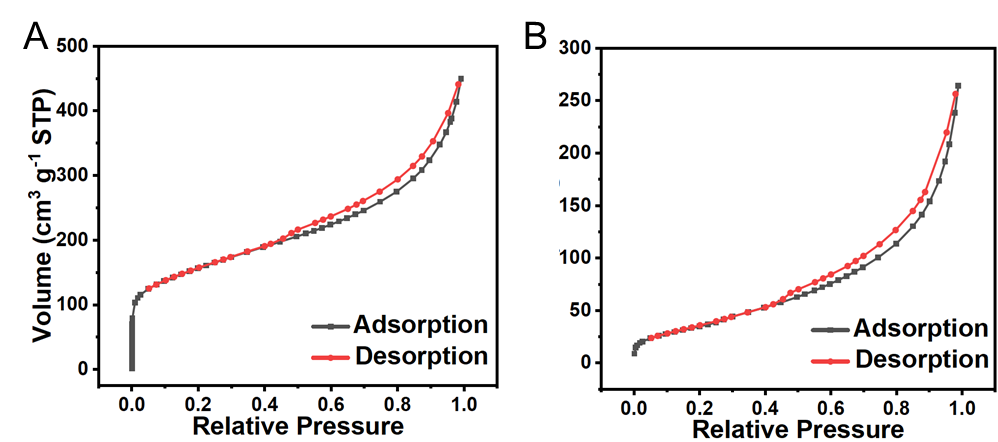


**Figure S7.** (a)N_2_ adsorption-desorption isotherm of OMG17. (b)N_2_ adsorption-desorption isotherm of OMG17@TiO_2_.

**Table S4.** Comparison of MB and RhB adsorption capacity of OMG17 and different materials.

| **Adsorbents** | **Materials and methods** | **Adsorption capacity for MB (mg/g)** | **Ref** |
| --- | --- | --- | --- |
| **OMG17** | Ball milling of spent graphite | 480.9 | This work |
| **OMG17@TiO_2_** | Ball milling of spent graphite | 673.67 | This work |
| TCGA-1 | Modified Hummer's method and hydrothermal reaction with thiourea | 101.5 | (1) |
| RGO | Modified Hummer's method and reduction via Fe | 111.6 | (2) |
| 3D graphene  aerogel | Modified Hummer's method and centrifuge vacuum evaporation | 397.0 | (3) |
| Granular activated carbon | Bituminous steam  activation | 263.8 | (4) |
| GO | Modified Hummer's method | 403.3 | (5) |
| F-GO | Modified Hummer's method and fluorination with HF | 306.5 | (5) |
| Graphite oxide | Ball milling of commercial graphite | 119.6 | (6) |
| Mesoporous exfoliated graphite | Microwave irradiation | 141.9 | (7) |

| **Adsorbents** | **Materials and methods** | **Adsorption capacity for RhB (mg/g)** | **Ref** |
| --- | --- | --- | --- |
| **OMG17** | Ball milling of spent graphite | 1015.9 | This work |
| **OMG17@TiO_2_** | Ball milling of spent graphite | 966.58 | This work |
| GB-3 | Improved Hummers' method | 64.5 | (8) |
| TA-G | Modified Hummers and Offeman method | 201.0 | (9) |
| GO/4A | Improved Hummers' method | 62.8 | (10) |
| Alg-GO-Lys | Microwave-activated amination | 178 | (11) |
| GO-PDA | Improved Hummers' method | 87.0 | (12) |
| PU/10GO membrane | Modified Hummer's method | 77.2 | (13) |
| Carboxy-GO/zeolite | Acid treatment and functionalize | 67.6 | (14) |

**Table S5.** Langmuir and Freundlich model of OMG17.

| **Langmuir isotherm model of MB** | | | | |
| --- | --- | --- | --- | --- |
| T(k) | Q_max_ (mg/g) | K_L_ (L/mg) | R^2^ |  |
| 298 | 480.93±10.37 | 24.21±3.98 | 0.97 |  |

| **Langmuir isotherm model of Rhodamine B** | | | | |
| --- | --- | --- | --- | --- |
| T(k) | Q_max_ (mg/g) | K_L_ (L/mg) | R^2^ |  |
| 298 | 342.52±45.64 | -0.06±0.027 | 0.70 |  |

**Table S6.** Fitted parameters of pseudo-first-order model and pseudo-first-order model of OMG17.

| **Kinetics model of MB** | | | |
| --- | --- | --- | --- |
| T (K) | Q_cal_ (mg/g) | K_1_ (min^-1^) | R^2^ |
| 298 | 442.58±10.1 | 0.089±0.012 | 0.95 |
| T (K) | Q_cal_ (mg/g) | K_2_ (g·mg^-1^·min^-1^) | R^2^ |
| 298 | 466.09±7.023 | 3.23×10^-4^±4.36×10^-5^ | 0.98 |

| **Kinetics model of Rhodamine B** | | | |
| --- | --- | --- | --- |
| T (K) | Q_cal_ (mg/g) | K_1_ (min^-1^) | R^2^ |
| 298 | 965.17±0.86 | 0.06±0.00079 | 0.88 |
| T (K) | Q_cal_ (mg/g) | K_2_ (g·mg^-1^·min^-1^) | R^2^ |
| 298 | 994.23±9.00 | 1.32×10^-4^±1.78×10^-5^ | 0.98 |

**Table S7.** Comparation of rate constant of different materials.

| **Adsorbents** | **Materials and methods** | **Rate constant for MB (g mg⁻¹ min⁻¹)** | **Ref** |
| --- | --- | --- | --- |
| **OMG17** | **Ball milling of spent graphite** | 0.000323507 | **This work** |
| TCGA-1 | Modified Hummer's method and hydrothermal reaction with thiourea | 0.1105 | (1) |
| RGO | Modified Hummer's method and reduction via Fe | 0.0013 | (2) |
| 3D graphene  aerogel | Modified Hummer's method and centrifuge vacuum evaporation | 0.0338 | (3) |
| GO | Modified Hummer's method | 0.008377 | (5) |
| F-GO | Modified Hummer's method and fluorination with HF | 0.003760 | (5) |


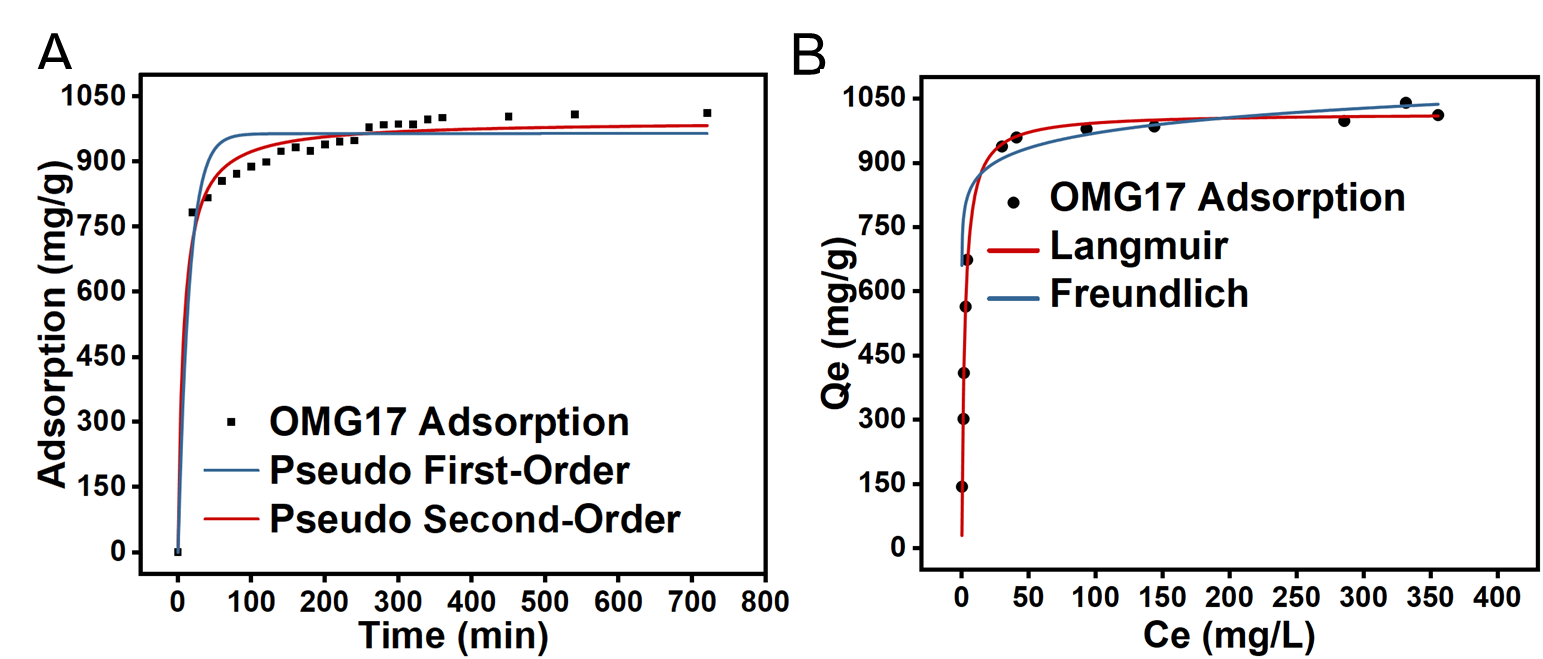


**Figure S8.** Langmuir model and quasi-second-order kinetic model of OMG17 (RhB).

**Table S8.** Langmuir and Freundlich model of OMG17@TiO_2_.

| **Langmuir isotherm model of MB** | | | | |
| --- | --- | --- | --- | --- |
| T(k) | Q_max_ (mg/g) | K_L_ (L/mg) | R^2^ |  |
| 298 | 673.67 | 2.30 | 0.98 |  |

| **Langmuir isotherm model of Rhodamine B** | | | | |
| --- | --- | --- | --- | --- |
| T(k) | Q_max_ (mg/g) | K_L_ (L/mg) | R^2^ |  |
| 298 | 966.58 | 0.608 | 0.98 |  |

**Table S9.** Fitted parameters of pseudo-first-order model and pseudo-first-order model of OMG17@TiO_2_.

| **Kinetics model of MB** | | | |
| --- | --- | --- | --- |
| T (K) | Q_cal_ (mg/g) | K_1_ (min^-1^) | R^2^ |
| 298 | 642.99±11.97 | 0.047±0.0060 | 0.95 |
| T (K) | Q_cal_ (mg/g) | K_2_ (g·mg^-1^·min^-1^) | R^2^ |
| 298 | 683.31±6.15 | 1.22×10^-4^±1.03×10^-5^ | 0.99 |

| **Kinetics model of Rhodamine B** | | | |
| --- | --- | --- | --- |
| T (K) | Q_cal_ (mg/g) | K_1_ (min^-1^) | R^2^ |
| 298 | 926.92±0.86 | 0.06±0.00073 | 0.89 |
| T (K) | Q_cal_ (mg/g) | K_2_ (g·mg^-1^·min^-1^) | R^2^ |
| 298 | 956.64±7.53 | 1.26×10^-4^±1.4×10^-5^ | 0.98 |


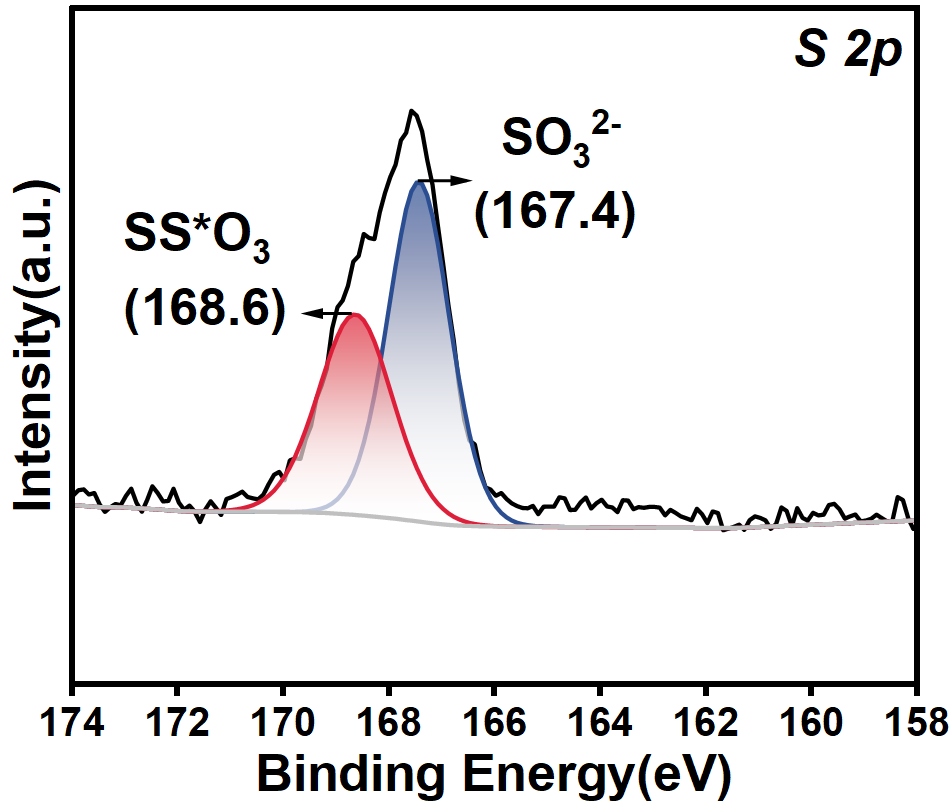


**Figure S9.** XPS S 2p of OMG17@TiO_2_.


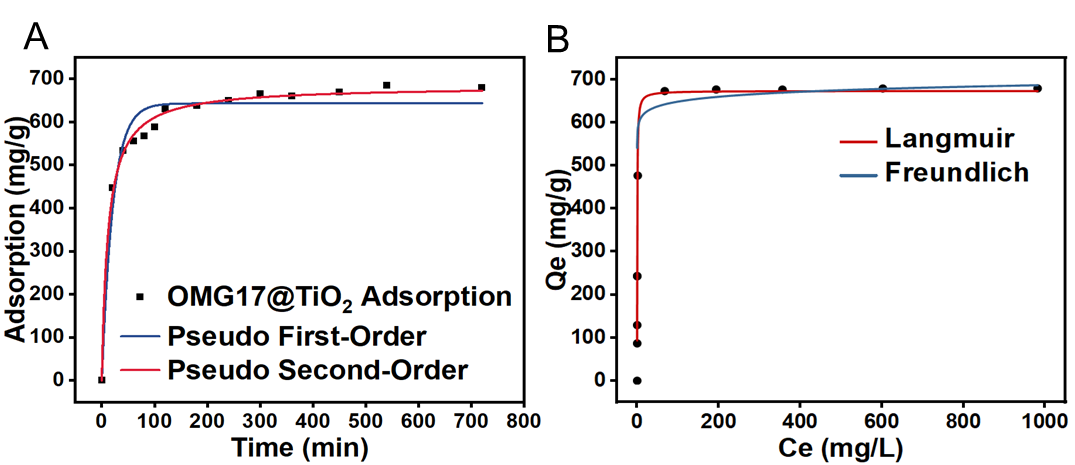


**Figure S10.** Langmuir model and quasi-second-order kinetic model of OMG17@TiO_2_ (MB).


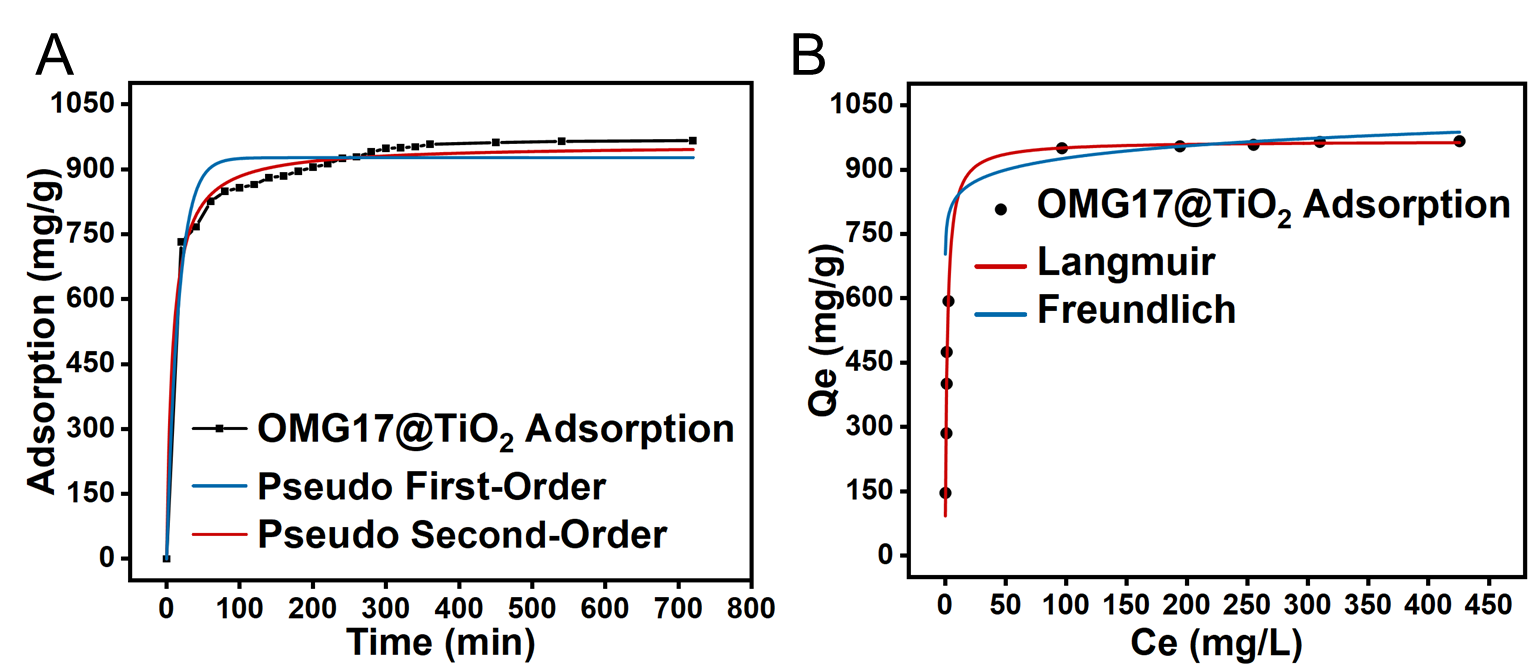


**Figure S11.** Langmuir model and quasi-second-order kinetic model of OMG17@TiO_2_ (RhB).


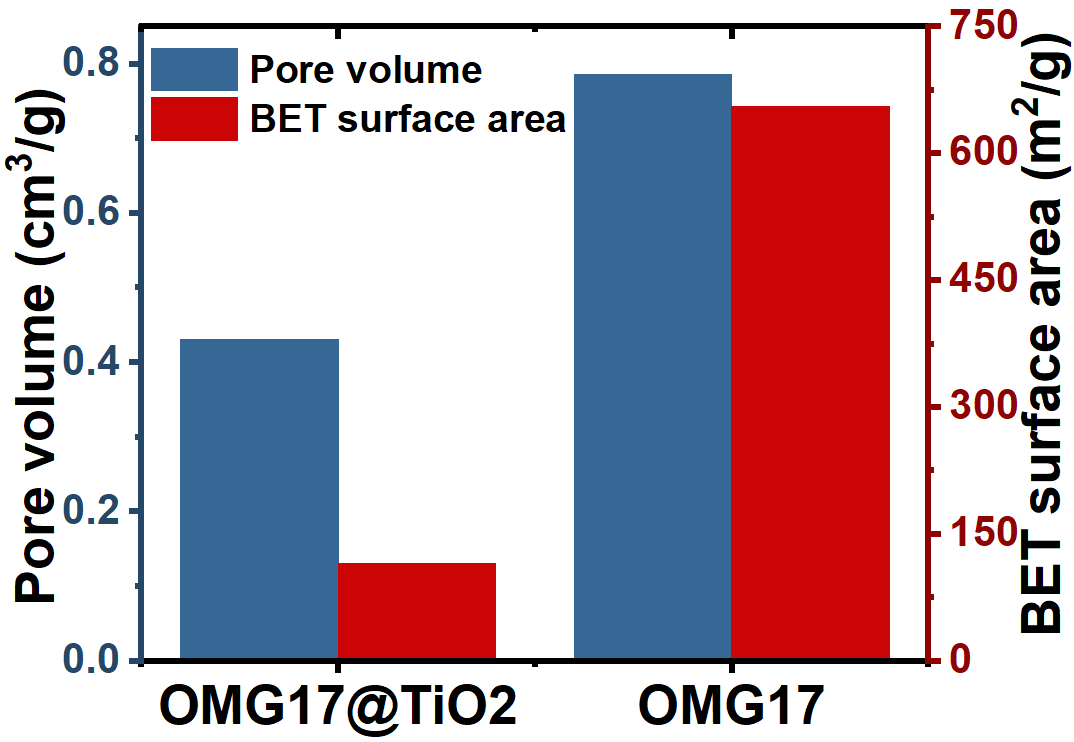


**Figure S12.** Decrease specific surface area and pore volume by electrostatic self-assembly process.


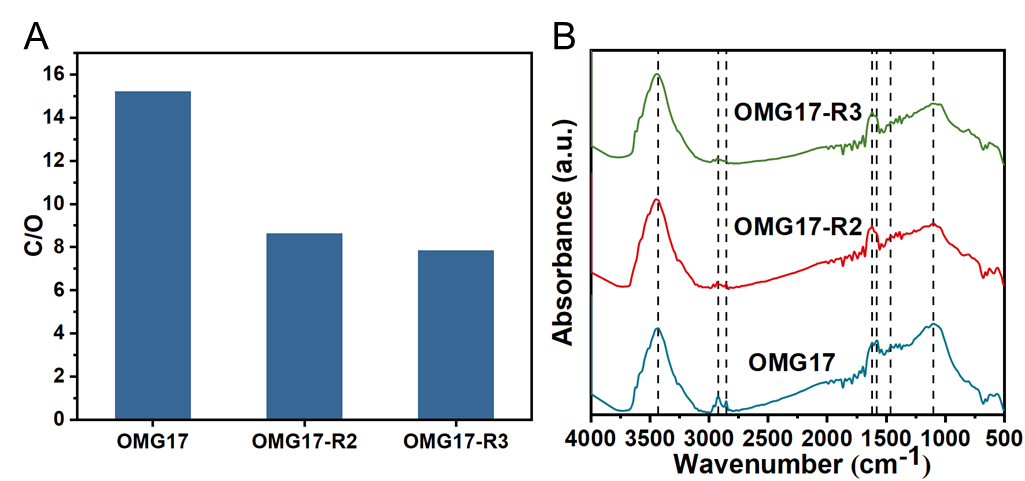


**Figure S13.** Comparison of (a) IR spectra; (b) C/O among OMG17 and cycles 2-3 times.

**Table S10.** Comparation of recovery efficiency and re-adsorption capacity of different materials.

| **Adsorbent** | **Synthesis method** | **Removal efficiency (%)** | **Re-adsorption capacity (mg g-1)** | **Ref.** |
| --- | --- | --- | --- | --- |
| **OMG17@TiO_2_** | **Ball mining** | **70** | **471.57** | **This work** |
| TiO_2_-graphene oxide(10wt%) | One-step colloidal blending | 90 | 33.53 | (15) |
| MnFe_2_O_4_-GO nanocomposite | Co-precipitation | 95 | 168.44 | (16) |
| CGO-AC/SA | Solvent aerogel | 87 | 156.04 | (17) |
| PVA/XG/GO | Freeze-thaw process | 87.9 | 254.3 | (18) |


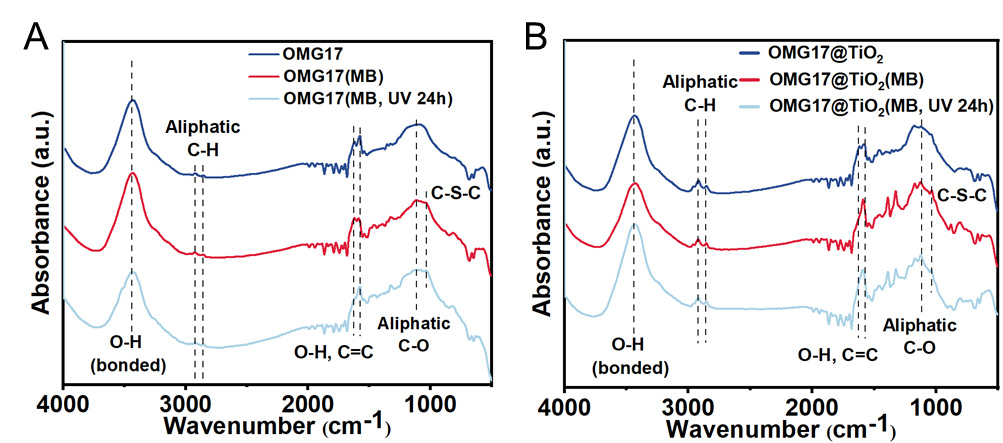


**Figure S14.** Different phase IR spectra of OMG17 and OMG17@TiO_2_.


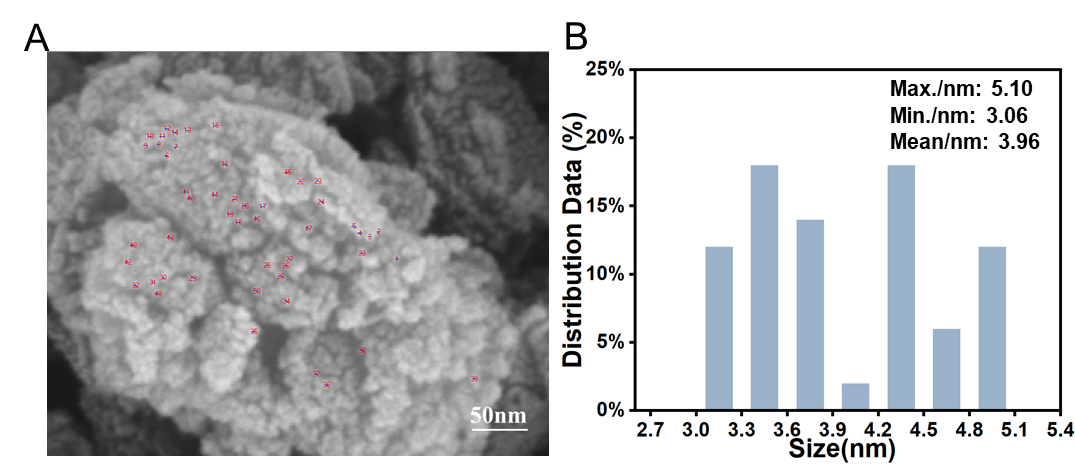


**Figure S15.** The particle size of methylene blue.


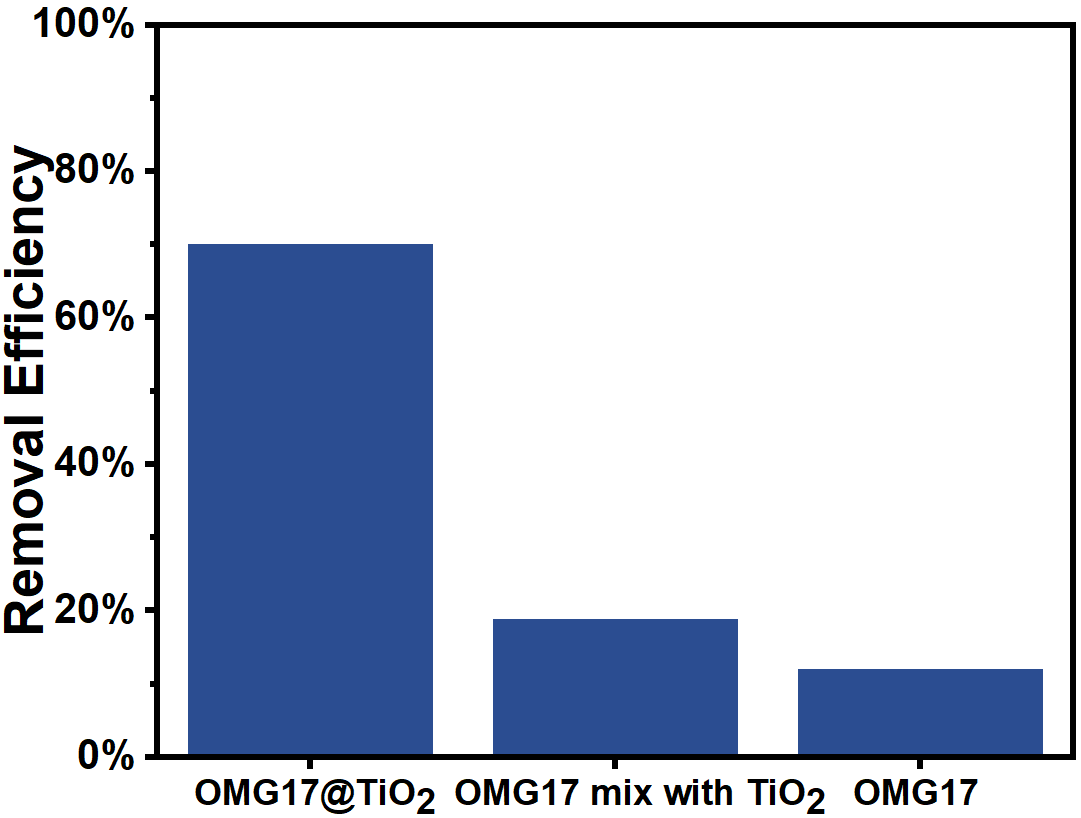


**Figure S16.** Comparation of UV regeneration efficiency of OMG17 and the mixture of OMG17 with TiO_2_.


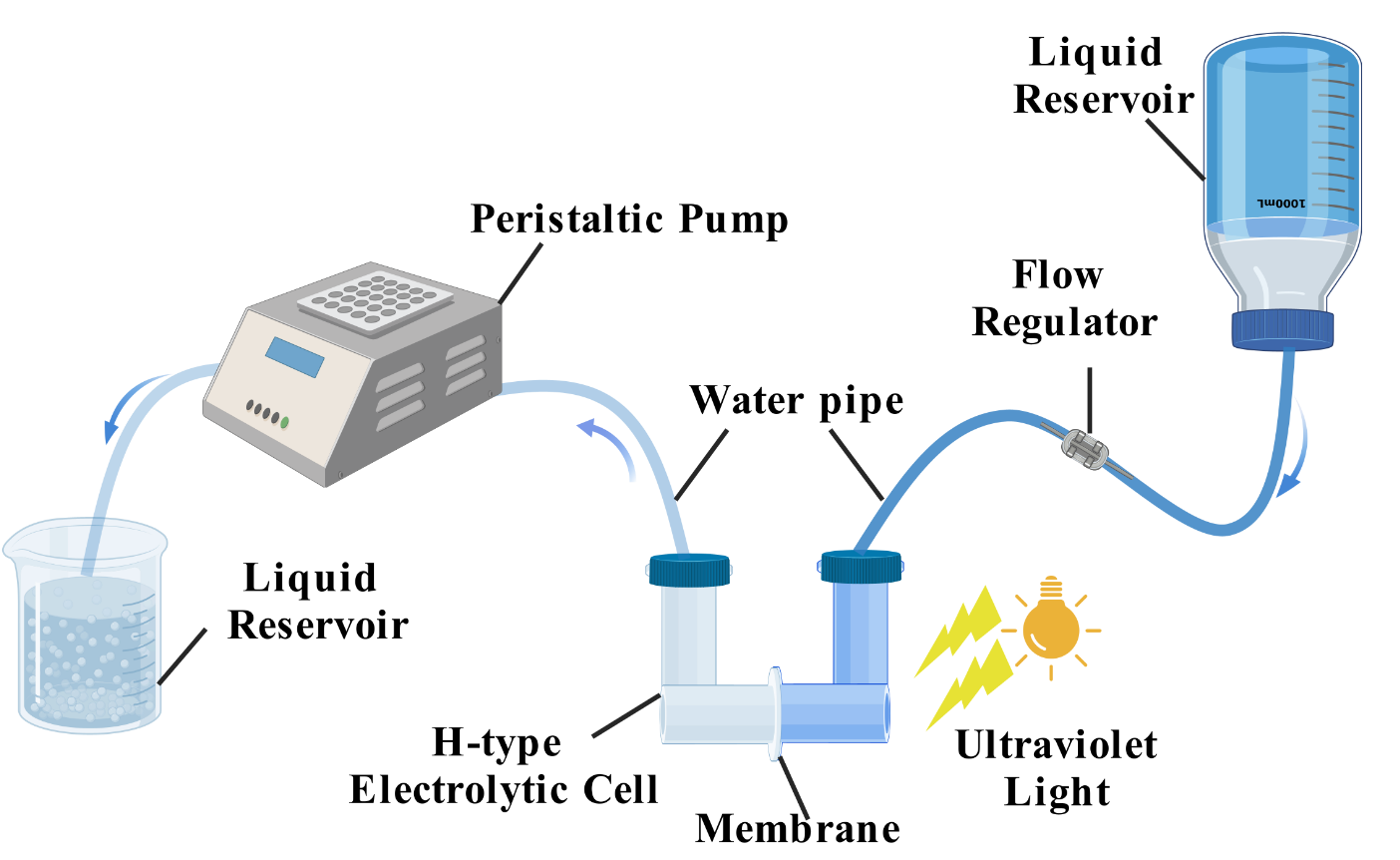


**Figure S17.** Test device for membrane filtration process.


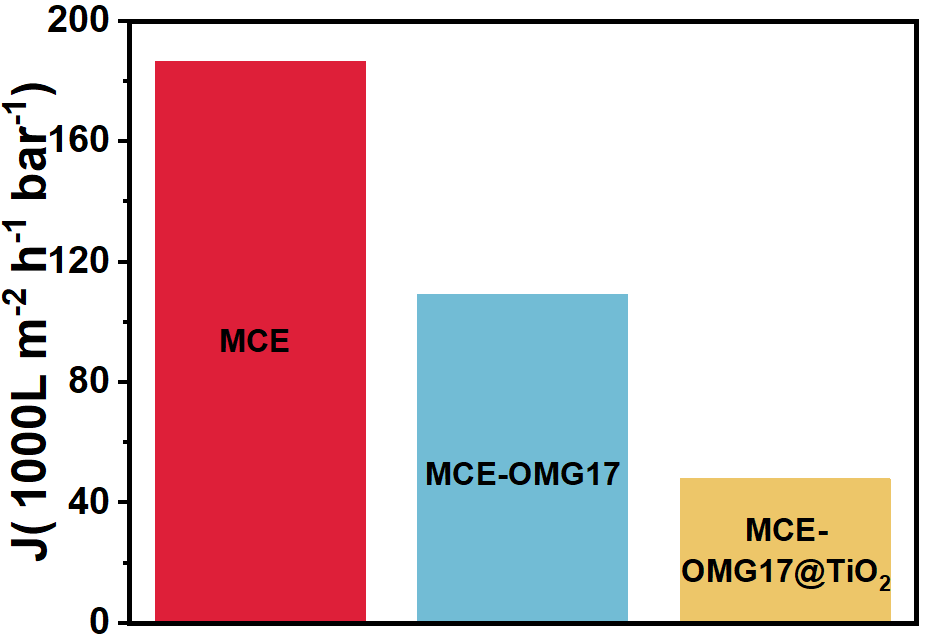


**Figure S18.**Water fluxes of membranes.

**
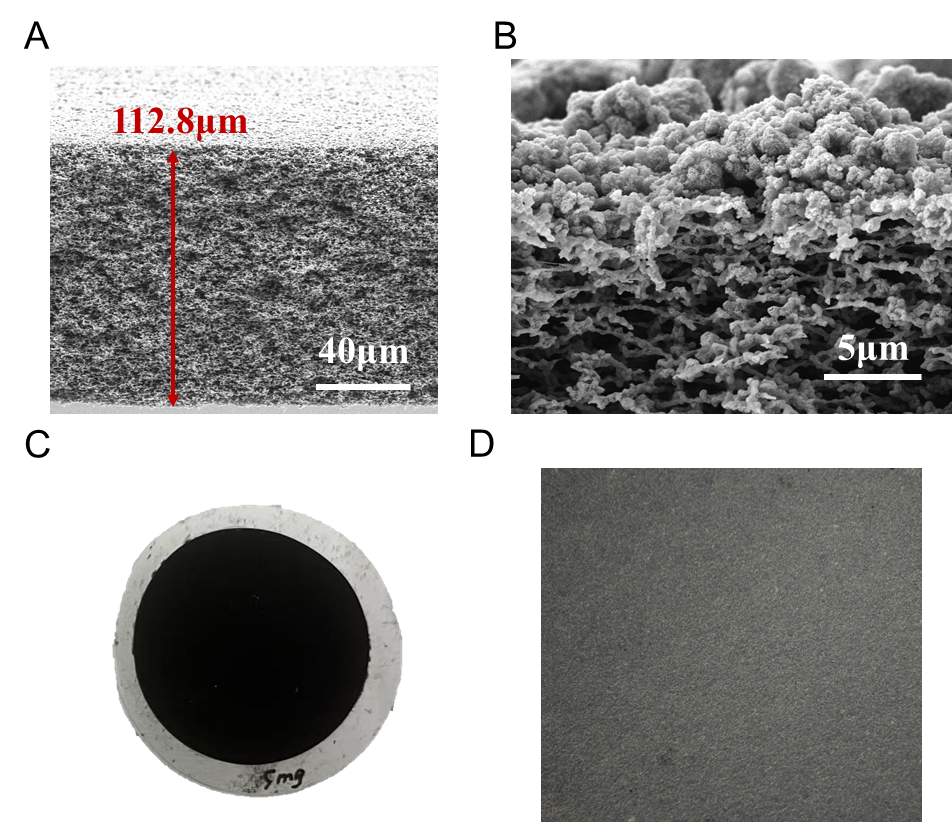
**

**Figure S19.** (a) SEM image of base membrane, (b) SEM image of OMG17@TiO_2_, (c) photo of the OMG17@TiO_2_ membrane, (d) photo of the OMG17@TiO_2_ membrane under a microscope.

**Table S11.** Performance parameters of the membrane.

|  | **Thickness (μm)** | **Tensile strength (MPa)** | **Elastic modulus(MPa)** |
| --- | --- | --- | --- |
| Base membrane | 112.8 | 3.2 | 64.46 |
| OMG17@TiO_2_ | 117.0 | 3.4 | 159.17 |

**Table S12.** Comparation of rate constant of different materials.

| **Raw material** | **This work** | **LME**(19) | **Hummers**(19) | **Cost** |
| --- | --- | --- | --- | --- |
| Waste graphite | 1 kg | Unused | Unused | ¥ 3 kg^-1^ |
| Flake graphite  FGP | Unused | Unused | 1 kg | ¥ 6 kg^-1^ |
|  | Unused | 1 kg | Unused | ¥ 36 kg^-1^ |
| H_2_SO_4_ | Unused | 1 kg | 30 kg | ¥ 1 kg^-1^ |
| KMnO_4_ | Unused | Unused | 3 kg | ¥ 18 kg^-1^ |
| Water | 21 kg | 1 kg | 100 kg | ¥ 0.004 kg^-1^ |
| H_2_O_2_ | 0.01 kg | Unused | 2 kg | ¥ 0.95 kg^-1^ |
| HCl | 0.036 kg | Unused | Unused | ¥ 500 kg^-1^ |
| Perfluoropolyether | Unused | Unused | 0.01 kg | ¥ 0.5 kg^-1^ |
| **Total** | **¥ 3.175 kg^-1^** | **¥ 26.77 kg^-1^** | **¥ 57.69 kg^-1^** |  |

| **Energy-consuming component** | **This work** | **LME**(15) | **Hummers**(15) | **Time** |
| --- | --- | --- | --- | --- |
| EC reaction power supply | Unused | 0.1275 kW | Unused | 48 h |
| Control and transmission systems | Unused | 0.8 kW | Unused | 48 |
| Stirring motor | Unused | Unused | 8 kW | 48 |
| Cooling-water machine | Unused | Unused | 3 kW | 12 |
| Automatic feeder. | Unused | Unused | 0.5 kW | 10 |
| Industrial ball mill | 0.75 kW | Unused | Unused | 48 |
| Cloth bag filter | 15 kW | Unused | Unused | 2.4 s |
| Drying machine | 20 kW | Unused | Unused | 28.8 s |
| Heating furnace | 35 kW | Unused | Unused | 60 s |
| **Total** | **36.75 kW** | **21.33 kW** | **75.75 kW** |  |

| **Carbon emission source** | **This work** | **LME**(15) | **Hummers**(15) | **Carbon emission factor** |
| --- | --- | --- | --- | --- |
| H_2_SO_4_ | Unused | 1 kg | 30 kg | 193.9kgCO2eq/t |
| KMnO_4_  Water | Unused | Unused | 3 kg | 193.9kgCO2eq/t |
|  | 21 kg | 1 kg | 100 kg | 193.9kgCO2eq/t |
| H_2_O_2_ | 0.01 kg | Unused | 2 kg | 193.9kgCO2eq/t |
| HCl | 0.036 kg | Unused | Unused | 193.9kgCO2eq/t |
| Perfluoropolyether | Unused | Unused | 0.01 kg | 193.9kgCO2eq/t |
| Electricity | 36.75 kW | 21.33 kW | 75.75 kW | 193.9kgCO2eq/kWh |
| **Total** | **19.78 kgCO2eq** | **11.74 kgCO2eq** | **43.42 kgCO2eq** |  |

**References**

1. Wang Y, Cui X, Wang Y, Shan W, Lou Z, Xiong Y. A thiourea cross-linked three-dimensional graphene aerogel as a broad-spectrum adsorbent for dye and heavy metal ion removal. New J. Chem. 2020;44(38):16285–16293.

2. Fan ZJ, Kai W, Yan J, Wei T, Zhi LJ, Feng J, Ren Y, Song LP, Wei F. Facile Synthesis of Graphene Nanosheets via Fe Reduction of Exfoliated Graphite Oxide. ACS. Nano. 2011;5(1):191–198.

3. Liu F, Chung S, Oh G, Seo TS. Three-Dimensional Graphene Oxide Nanostructure for Fast and Efficient Water-Soluble Dye Removal. ACS Appl. Mater. Interfaces. 2012;4(2):922–927.

4. Allgayer R, Yousefi N, Tufenkji N. Graphene oxide sponge as adsorbent for organic contaminants: comparison with granular activated carbon and influence of water chemistry. Environ. Sci.: Nano. 2020;7(9):2669–2680.

5. Mao B, Sidhureddy B, Thiruppathi AR, Wood PC, Chen A. Efficient dye removal and separation based on graphene oxide nanomaterials. New J. Chem. 2020;44(11):4519–4528.

6. Mahmoud AED, Stolle A, Stelter M. Sustainable Synthesis of High-Surface-Area Graphite Oxide via Dry Ball Milling. ACS Sustainable Chem. Eng. 2018;6(5):6358–6369.

7. Saikam L, Arthi P, Jayram ND, Sykam N, Rapid removal of organic dyes from aqueous solutions using mesoporous exfoliated graphite. *Diamond and Related Materials.* 2022;**130**:109480.

8. Cheng ZL, Li YX, Liu Z. Novel adsorption materials based on graphene oxide/Beta zeolite composite materials and their adsorption performance for rhodamine B. Journal of Alloys and Compounds. 2017;708:255–263.

9. Liu K, Li H, Wang Y, Gou X, Duan Y. Adsorption and removal of rhodamine B from aqueous solution by tannic acid functionalized graphene. Colloids and Surfaces A: Physicochemical and Engineering Aspects. 2015;477:35–41.

10. Liu X, Guo Y, Zhang C, Huang X, Ma K, Zhang Y. Preparation of graphene oxide/4A molecular sieve composite and evaluation of adsorption performance for Rhodamine B. Separation and Purification Technology. 2022;286:120400.

11. Tunioli F, Khaliha S, Mantovani S, Bianchi A, Kovtun A, Xia Z, Bafqi MSS, Okan BS, Marforio TD, Calvaresi M, et al. Adsorption of emerging contaminants by graphene related materials and their alginate composite hydrogels. Journal of Environmental Chemical Engineering. 2023;11(2):109566.

12. Wang X, Guo Y, Jia Z, Ma H, Liu C, Liu Z, Shi Q, Ren B, Li X, Zhang X, et al. Fabrication of graphene oxide/polydopamine adsorptive membrane by stepwise in-situ growth for removal of rhodamine B from water. Desalination. 2021;516:115220.

13. Sundaran SP, Reshmi CR, Sagitha P, Manaf O, Sujith A. Multifunctional graphene oxide loaded nanofibrous membrane for removal of dyes and coliform from water. Journal of Environmental Management. 2019;240:494–503.

14. Yu Y, Murthy BN, Shapter JG, Constantopoulos KT, Voelcker NH, Ellis AV. Benzene carboxylic acid derivatized graphene oxide nanosheets on natural zeolites as effective adsorbents for cationic dye removal. Journal of Hazardous Materials. 2013;260:330–338.

15. Nauyen-Phan TD, Pham VH, Shin EW, Pham HD, Kim S, Chung JS, Kim EJ, Hur SH. The role of graphene oxide content on the adsorption-enhanced photocatalysis of titanium dioxide/graphene oxide composites. Chemical Engineering Journal. 2011;170(1):226-232.

16. Huong PTL, Tu N, Lan H, Thang LH, Quy NV, Tuan PA, Dinh NX, Phan VN, Le AT. Functional manganese ferrite/graphene oxide nanocomposites: effects of graphene oxide on the adsorption mechanisms of organic MB dye and inorganic As(v) ions from aqueous solution. RSC Advances. 2018;8(22):12376-12389.

17. Zhao J, Liu S, Lai Q, Liu L, Gao Y. Functional carboxylated graphene oxide aerogel spheres for high-efficiency cationic dye adsorption: Synergistic design, mechanism, and regeneration. Desalination. 2025;614:119204.

18. Zhang M, Xue Y, Zhou H, Xiang A, Deng Y. Adsorption behaviors and mechanisms of polyvinyl alcohol/xanthan gum/graphene oxide porous hydrogel for methylene blue and congo red. International Journal of Biological Macromolecules. 2025;308:142662.

19. Guo J, Pei S, Huang K, Zhang Q, Zhou X, Tong J, Liu HM, Ren W. Control of water for high-yield and low-cost sustainable electrochemical synthesis of uniform monolayer graphene oxide. Nat. Commun. 2025;16(1):727.
